# Synaptic Vesicle Glycoprotein 2A: Features and Functions

**DOI:** 10.3389/fnins.2022.864514

**Published:** 2022-04-28

**Authors:** Rachele Rossi, Shokouh Arjmand, Simone Larsen Bærentzen, Albert Gjedde, Anne M. Landau

**Affiliations:** ^1^Translational Neuropsychiatry Unit, Department of Clinical Medicine, Aarhus University, Aarhus, Denmark; ^2^Department of Nuclear Medicine and PET Center, Aarhus University Hospital, Aarhus, Denmark; ^3^Department of Neuroscience, University of Copenhagen, Copenhagen, Denmark; ^4^Department of Neurology and Neurosurgery, McGill University, Montreal, QC, Canada

**Keywords:** synaptic vesicle glycoprotein 2A (SV2A), synaptic density, levetiracetam, neuroimaging, synaptic vesicles, positron emission tomography (PET), UCB-J

## Abstract

In recent years, the field of neuroimaging dramatically moved forward by means of the expeditious development of specific radioligands of novel targets. Among these targets, the synaptic vesicle glycoprotein 2A (SV2A) is a transmembrane protein of synaptic vesicles, present in all synaptic terminals, irrespective of neurotransmitter content. It is involved in key functions of neurons, focused on the regulation of neurotransmitter release. The ubiquitous expression in gray matter regions of the brain is the basis of its candidacy as a marker of synaptic density. Following the development of molecules derived from the structure of the anti-epileptic drug levetiracetam, which selectively binds to SV2A, several radiolabeled markers have been synthetized to allow the study of SV2A distribution with positron emission tomography (PET). These radioligands permit the evaluation of *in vivo* changes of SV2A distribution held to be a potential measure of synaptic density in physiological and pathological conditions. The use of SV2A as a biomarker of synaptic density raises important questions. Despite numerous studies over the last decades, the biological function and the expressional properties of SV2A remain poorly understood. Some functions of SV2A were claimed, but have not been fully elucidated. While the expression of SV2A is ubiquitous, stronger associations between SV2A and Υ amino butyric acid (GABA)-ergic rather than glutamatergic synapses were observed in some brain structures. A further issue is the unclear interaction between SV2A and its tracers, which reflects a need to clarify what really is detected with neuroimaging tools. Here, we summarize the current knowledge of the SV2A protein and we discuss uncertain aspects of SV2A biology and physiology. As SV2A expression is ubiquitous, but likely more strongly related to a certain type of neurotransmission in particular circumstances, a more extensive knowledge of the protein would greatly facilitate the analysis and interpretation of neuroimaging results by allowing the evaluation not only of an increase or decrease of the protein level, but also of the type of neurotransmission involved.

## Introduction

The number of studies of synaptic vesicle glycoprotein 2A (SV2A) rose exponentially after the discovery of an anti-epileptic drug, levetiracetam, that selectively binds to SV2A. SV2A is a transmembrane protein of synaptic vesicles, ubiquitously expressed throughout the brain. The development of radiotracers that specifically target the protein proceeded from the structure of levetiracetam, inspired by the claim that SV2A is a suitable marker of synaptic density.

Until recently, synaptic density measurements were assessed only *in vitro*, generally with immunohistochemistry or electron microscopy. The synaptic vesicle protein synaptophysin traditionally was used as the ‘gold standard’ marker of pre-synaptic terminals, as it is one of the most abundant proteins of synaptic vesicles (Takamori et al., 2006). Synaptophysin immunoreactivity commonly is used to quantify synapses *post mortem* [e.g., detection of synaptic density loss in dementias with synaptophysin immunohistochemistry, reviewed by Clare et al. (2010)].

Finnema et al. (2016) compared the regional densities of synaptophysin and SV2A, identifying a linear correlation between the two proteins in gray matter areas and suggesting the use of SV2A as a synaptic density marker as an alternative to synaptophysin. The development of SV2A tracers allowed the non-invasive evaluation of changes in synaptic density *in vivo* by PET, which is particularly relevant to brain disorders.

Despite extensive studies, much remains to be understood about the protein, and further experimentation could lead to the discovery of new SV2A functions with implications for disease research and interpretation of neuroimaging results. In this review, we summarize the findings of the available literature about SV2A features and functions, and we evaluate its utilization in neuroimaging as a marker of synaptic density, especially in studies of neurodegenerative disorders. Furthermore, we report interesting and incompletely understood facts about the protein, with the purpose of raising new questions for future research.

## Synaptic Vesicle Glycoprotein 2 Family Structures and Functions

### Synaptic Vesicle Glycoprotein 2 Family

The synaptic vesicle glycoproteins 2 (SV2) are keratan sulfate proteoglycans (Scranton et al., 1993) that constitute a family of proteins present in the secretory vesicles of neurons and endocrine cells (Buckley and Kelly, 1985; Portela-Gomes et al., 2000), consistent with their fundamental role in vesicular dynamics.

Adopting an approach that allowed the isolation and analysis of single synaptic vesicles representative of all neurotransmitter classes, Mutch et al. (2011) found that each synaptic vesicle contains on average 5.04 copies of SV2 proteins, with only a small intervesicular variability. However, in an earlier mass spectrometry study, Takamori et al. (2006) estimated only 1.7 SV2 copies per synaptic vesicle.

The importance of the SV2 family is suggested also by the sequencing of cDNA clones encoding SV2 from evolutionary distant species, which shows the high conservation of SV2 proteins throughout evolution and across eukaryotic species (Bindra et al., 1993). The lack of SV2 homologs in yeast suggests that these proteins exert their functions in eukaryotic vesicular dynamics and possibly are not involved in the very basic machinery of secretion common to all species.

A first study (Bajjalieh et al., 1992), based on the deduced SV2 sequence, revealed that SV2 proteins contain 12 transmembrane domains and their NH2-terminal amino acid sequence resembles a family of bacterial transporters, suggesting a possible role of SV2 proteins as transmembrane transporters. Later (Jacobsson et al., 2007), a phylogenic analysis showed that SV2 proteins are, among the vertebrate proteins, the most similar to the solute carrier family 22, that belongs to the major facilitator superfamily.

SV2 proteins are highly glycosylated, with luminal glycosylation sites (Scranton et al., 1993); the N-glycosylation plays a only partially dispensable role in the sorting of SV2 in synaptic vesicles (Kwon and Chapman, 2012). In the synaptic vesicles of the electric organ of the animal model Torpedo, the glycosylation in the luminal domains of SV2 together with the glycidic parts of other synaptic vesicle proteins may collaborate to create a “vesicular matrix” able to capture acetylcholine and adenosine triphosphate (ATP), thus regulating their availability (Reigada et al., 2003).

The SV2 family has 3 members, SV2A, SV2B, and SV2C, that are similar in structure but different in pattern of expression. SV2A is the predominant isoform, ubiquitously present in all brain regions, with peaks in the subcortical areas such as basal ganglia and thalamus. In contrast, SV2B preferentially is expressed in cortex and hippocampus (Bajjalieh et al., 1993). The ubiquitous expression of SV2A was recently confirmed by autoradiography; the protein is distributed in all gray matter structures, with variable levels of expression (Varnäs et al., 2020). Neurons predominantly express one isoform but the expression patterns of SV2A and SV2B partly overlap, and some neurons such as the pyramidal neurons of hippocampus can express both isoforms; sometimes, both isoforms also are co-expressed in the same synaptic vesicle. It is remarkable that the expression patterns of the two isoforms appear not to correlate with the neurotransmitter content of the vesicles, nor with the expression patterns of other synaptic proteins. These findings suggest that SV2A and SV2B may perform same or similar functions in vesicular dynamics (Bajjalieh et al., 1994). The SV2C subtype structure is very similar to that of other isoforms, but the distribution is very restricted, with high levels in phylogenetically older brain areas such as striatum, substantia nigra, pons, medulla oblongata, and olfactory bulb, and close to absence from neocortex, hippocampus and thalamus (Janz and Südhof, 1999). A later study with fluorescent immunohistochemistry and *in situ* hybridization revealed particular expression of SV2C in striatal cholinergic interneurons and nigro-striatal mesolimbic dopaminergic neurons (Dardou et al., 2011).

The expression of SV2 isoforms varies during brain development, with possible effects on synaptic organization. SV2A fluctuations were observed in different telencephalic areas of the developing mouse brain, especially in the hippocampus (Crèvecoeur et al., 2013). SV2B expression is greater during development than in adult brain, especially in some structures of the basal ganglia (Bajjalieh et al., 1994). Interestingly, in the developing mouse retina, a temporal and cell-specific distribution of each SV2 isoform was detected in early developmental stages, prior to onset of synaptogenesis, indicating a possible synapse-specific role of the isoforms in synapse formation (Wang et al., 2003).

The biological significance of SV2 family members is unclear, although the participation in key mechanisms of the vesicular processes is widely accepted. However, based on the protein structures and properties, multiple specific roles were tested, including transport activity, interaction with synaptotagmin and its retrieval during vesicular recycling, participation in neurotransmission, with regulation of vesicular calcium sensitivity and vesicular priming, modulation of the readily releasable pool of vesicles, and interaction with the extracellular matrix. SV2 proteins also are responsible for the vesicular entry of neurotoxins and could possibly have a mitochondrial function (Table 1).

**TABLE 1:** Possible functions of synaptic vesicle glycoproteins 2 (SV2).

Functions	Main analyzed SV2 isoform	Main references
Transport of galactose	SV2A	Madeo et al. (2014)
Interaction with synaptotagmin	SV2A	Schivell et al. (1996), Lambeng et al. (2006)
	SV2B	Lazzell et al. (2004)
	SV2A/SV2B	Pyle et al. (2000)
	SV2A/SV2C	Schivell et al. (2005)
Regulation of synaptotagmin trafficking and expression	SV2A	Nowack et al. (2010), Yao et al. (2010), Zhang et al. (2015)
Regulation of neurotransmitters release	SV2A	Crowder et al. (1999), Bradberry and Chapman (2021)
	SV2A/SV2B	Janz et al. (1999)
Involvement in a maturation step of vesicles during calcium-induced exocytosis	SV2A	Xu and Bajjalieh (2001)
	SV2A/SV2B	Custer et al. (2006), Chang and Südhof (2009)
Modulation of dimensional adaptations of vesicles	SV2A	Budzinski et al. (2009)
Interaction with laminin	Not specified	Son et al. (2000)
Mediation of cellular entrance of botulinum and tetanus neurotoxins	SV2A/SV2B/SV2C	Dong et al. (2006), Rummel et al. (2009), Peng et al. (2011), Blum et al. (2012), Mahrhold et al. (2013), Weisemann et al. (2016), Yao et al. (2016)
	SV2A/SV2B	Dong et al. (2008), Yeh et al. (2010), Kroken et al. (2011)
	SV2C	Gustafsson et al. (2018)
	Not specified	Fu et al. (2009), Colasante et al. (2013)
Mitochondrial role as fusion or fission factor	SV2A	Stockburger et al. (2016)
Interaction with adenine nucleotides	SV2A/SV2B	Yao and Bajjalieh (2008)
Interaction with synaptic vesicle proteins	SV2A	Wittig et al. (2021)

### Synaptic Vesicle Glycoproteins 2 as Transporters

Along with a similarity to bacterial transporters (Bajjalieh et al., 1992), SV2 proteins present a homology of 6 C-terminal transmembrane domains with plasma membrane neurotransmitter transporters. For this reason, SV2 proteins were initially thought to mediate the transport of neurotransmitters into synaptic vesicles (Feany et al., 1992). Thus, the discovery of the different distribution of SV2 isoforms initially was held to be related to the possibility that each SV2 subtype (particularly SV2A and SV2B) would be responsible for the transport of different amino acid neurotransmitters, such as the excitatory glutamate or the inhibitory GABA. However, as stated above, their patterns of expression are inconsistent with transport of specific neurotransmitters. For example, in the cerebellum, in which excitatory and inhibitory cells are well defined, SV2A appears to be expressed in both glutamatergic and GABAergic neurons, while SV2B is present in most but not all glutamatergic neurons and absent from GABAergic neurons (Bajjalieh et al., 1994). As a result, currently there is no experimental evidence for the hypothesis that SV2 proteins have a role as neurotransmitter transporters.

Remarkably, the SV2A subtype was shown to mediate the transport of a particular substrate, galactose. In a model of hexose transporter-deficient *Saccharomyces cerevisiae*, the induced expression of human SV2A allowed the growth of the yeast on a galactose-containing medium, but not on other fermentable carbon sources, possibly demonstrating the role and specificity of SV2A as galactose transporter, and further evidence emerged with the direct measurement of galactose uptake (Madeo et al., 2014).

This putative function of SV2A deserves attention and further analysis, as it may underlie specific roles of galactose within neurons and may help to understand the implication of SV2A in pathological contexts.

### Synaptic Vesicle Glycoproteins 2 Interact With Synaptotagmin

SV2 proteins bind synaptotagmin that has a fundamental role in the regulation of neurotransmitter release as a calcium sensor active during exocytosis. The interaction between synaptotagmin-I and the predominant isoform SV2A was the first to be investigated, showing the binding to be specific, mediated by the cytosolic amino terminus of SV2A, and calcium-dependent. The binding is inhibited by increased calcium concentrations, probably because the interaction is regulated by calcium-induced conformational changes of synaptotagmin (Schivell et al., 1996). SV2B also interacts with synaptotagmin, but with different properties and apparently in a calcium-independent manner (Lazzell et al., 2004).

A consequent study (Schivell et al., 2005) demonstrated that all SV2 isoforms present the same synaptotagmin binding site inhibitable by calcium, while the isoforms SV2A and SV2C contain an additional modulatory calcium-induced binding site in the amino terminus. The authors suggested that SV2 proteins may control synaptotagmin interaction with other proteins to modulate vesicular exocytosis, and that the SV2B isoform may work differently from SV2A and SV2C because of the presence of a single synaptotagmin interaction site. Solubilization and immunoprecipitation of the proteins confirmed the binding between the isoform SV2A and synaptotagmin (Lambeng et al., 2006).

SV2 proteins also appear to have a role in controlling the abundance of synaptotagmin in synaptic vesicles; in fact, the mutation of a fundamental tyrosine-based endocytosis motif in SV2A reduces the trafficking of synaptotagmin in synaptic vesicles. Moreover, in neurons knocked-out for both SV2A and SV2B, synaptotagmin is greatly reduced in vesicles and as a consequence fewer vesicles are competent to achieve the calcium-mediated fusion (Yao et al., 2010).

Phosphorylation of SV2 proteins plays a role in these processes. SV2 molecules are phosphoproteins of the nerve terminals, where the phosphorylation of the amino terminus seems to play a crucial role in mediating the interaction with synaptotagmin, as the phosphorylation increases the affinity of SV2 for synaptotagmin (Pyle et al., 2000). Casein kinase 1 family members are able to phosphorylate the SV2A isoform in two groups of residues, called Cluster-1 and Cluster-2. The phosphorylation of a particular residue (Thr84) belonging to Cluster-2 is important as a trigger of the binding of SV2A to synaptotagmin-I. In addition, the ablation of Thr84 phosphorylation in SV2A results in an erroneous retrieval during vesicular endocytosis of synaptotagmin-I. Notably, SV2A phosphorylation is necessary for synaptotagmin sorting but not for endocytosis in general (Zhang et al., 2015).

Overall, SV2 proteins have a major role in regulating the presence and abundance of synaptotagmin in synaptic vesicles, by modulating its trafficking to the vesicles and its retrieval during endocytosis.

Nowack et al. (2010) found that the influence on synaptotagmin’s expression and retrieval is a fundamental function of SV2 members in the synapse, supported by functional motifs of the proteins that are different from those regulating the probability of neurotransmitter release. In primary neuronal cultures from SV2A/SV2B double knock-out mice, the authors performed mutagenic analysis of tryptophans W300 and W666, conserved among all the SV2 isoforms. Remarkably, the authors found that mutants SV2A-W300A and SV2A-W666A are unable to restore normal neurotransmission, but do restore normal levels of synaptotagmin expression and internalization. These results indicate that the residues W300 and W666 do not affect the dynamics of synaptotagmin in the synapse but rather play a crucial role in neurotransmission, demonstrating that SV2 proteins perform at least two roles in synapses, the roles exerted by different functional motifs (Nowack et al., 2010).

### Synaptic Vesicle Glycoproteins 2 Regulate the Release of Neurotransmitters

Although SV2 proteins probably are not involved in neurotransmitter transport, their fundamental role in synaptic function is evident. Studies of SV2A mutants have assessed the importance of the protein in neurotransmission. In the study by Janz et al. (1999), SV2A-deficient mice had impaired and abnormal neural activity, and did not survive past the first weeks of life. In addition, SV2A/SV2B double knock-out mutants had properties similar to SV2A mutants, suggesting that the knock-out of SV2B does not aggravate the phenotype. As discussed by the authors, this result can be explained with the distribution patterns of the isoforms, as SV2A is more widely expressed than SV2B, potentially making its deletion more significant. Notably, analysis of the levels of key synaptic proteins in SV2 mutants tested whether the deficits relate to changes of synaptic abundance and composition and revealed no changes of synaptic vesicle abundance and/or gross brain morphology (Janz et al., 1999). Crowder et al. (1999) confirmed the consequences of SV2A knock-out. Homozygous mutant mice lacking the expression of SV2A appeared normal at birth, but then revealed a severe phenotype with growth failure, presence of seizures (thought possibly to be associated with reduced inhibitory transmission in hippocampus) and death within the third week of post-natal life. Again, SV2A knock-down seemed not to be associated with alterations of brain morphology, nor of synaptic density (Crowder et al., 1999).

The interaction between SV2 proteins and the calcium-sensor synaptotagmin may explain how SV2 proteins are involved in neurotransmission, as the calcium concentration is a key factor in vesicular fusion. However, the relationship between SV2 proteins and calcium remains complicated. Janz et al. (1999) proposed a direct involvement of SV2 proteins in the regulation of intracellular calcium as calcium transporters. This hypothesis comes from the observation that SV2 knock-out results in pre-synaptic calcium accumulation and increased release probability, likely involved in the development of seizures. However, Iezzi et al. (2005) later rejected the hypothesis that SV2 proteins are calcium transporters: up- or down-regulation of the isoforms SV2A and SV2C does not change cytosolic or intravesicular calcium levels, nor the calcium currents in pancreatic endocrine cells (Iezzi et al., 2005).

The way in which SV2 proteins are involved in neurotransmission is a complicated topic. In the study of Chang and Südhof (2009), the authors presented the possibility of a maturation step during exocytosis, previously unidentified, in which SV2 proteins render the primed vesicles in a fully calcium-responsive state. This means that SV2 proteins modulate neurotransmitter release in early stages of exocytosis, without affecting its final step. Importantly, in this study the authors demonstrated that the N-terminal sequence of SV2A that binds to synaptotagmin is not necessary for SV2A function, as a SV2A mutant lacking this motif fully can rescue the SV2-deficient phenotype, implying that the binding to synaptotagmin may not be functionally essential to a role of SV2 in exocytosis (Chang and Südhof, 2009).

A modulatory role in neurotransmitter release also is supported by the observation that the SV2A isoform is involved in dimensional modifications of the synaptic vesicles during neurotransmitter loading. Budzinski et al. (2009) reported that glutamatergic vesicles change significantly with respect to surface and volume upon filling with neurotransmitters, but the changes do not happen in vesicles that do not express SV2A. However, glutamate loading is possible in SV2A knock-out mice, indicating that SV2A has no role in processes of neurotransmitter loading itself, but only in the consequent dimensional adaptations of the vesicles, or, perhaps, that SV2A may influence the activity of other proteins involved in vesicle size changes. These findings also rejected the hypothesis that SV2 proteins are transporters of neurotransmitters. Given that loss of SV2A reduces the release probability, and that absence of SV2A prevents dimensional increase of vesicles filled with neurotransmitters, the authors suggested that the swelling of vesicles could be an intrinsic signal indicating that the vesicles are ready to be released (Budzinski et al., 2009).

Recently, the role of SV2A in calcium-induced exocytosis was evaluated by a new chemogenetic approach described by Bradberry and Chapman (2021), which allows the detection of both calcium entry and neurotransmitter release at the same pre-synaptic terminal. In hippocampal neurons lacking the expression of SV2A, the probability of neurotransmitter release declined without alterations of calcium influx, indicating that SV2A probably can increase the likelihood that calcium induces an event of vesicular fusion. These results confirm the modulatory action of SV2 proteins in exocytosis, and suggest a role for SV2A in excitation-secretion coupling (Bradberry and Chapman, 2021).

The hypothesis that SV2 proteins mediate a maturation step of the calcium-induced exocytosis is supported by other studies. Xu and Bajjalieh (2001) demonstrated that SV2 proteins are required to maintain the pool of vesicles ready to be released during calcium-induced exocytosis. The calcium-induced exocytosis goes through an initial rapid phase called exocytotic burst, in which the docked vesicles fuse with the plasma membrane. The exocytotic burst operationally defines the readily releasable pool (RRP) of vesicles. In SV2A knock-out chromaffin cells, the exocytotic burst was significantly decreased, but the decrease was not attributable to a reduction in number or size of the RRP vesicles, nor to changes in calcium dependency of the fusion process. Further analyses revealed that the reduction in the exocytotic burst is given by SV2A’s capability to modulate the concentration and organization of pre-fusion complexes, such as components of the SNARE complex. In other words, SV2 proteins (and the SV2A isoform in particular) guide the vesicles of RRP in a fusion-competent state (Xu and Bajjalieh, 2001). Consistent with these findings, Chang and Südhof (2009) did not detect a decrease in the RRP size after SV2 protein deletion.

Other studies support the hypothesis that SV2 proteins exert their action in a maturation step that possibly occurs after the docking, and before the final event of vesicle fusion. This observation was reported in a study by Custer et al. (2006), showing that loss of SV2 proteins is associated with reduced initial release probability and decreased post-synaptic currents; in this case, the reduction is associated with a smaller RRP of vesicles. Alteration of the RRP does not reflect reduction of RRP refilling or altered release probability of single vesicles. Ultrastructural analyses demonstrated that the number of docked vesicles is unaltered in neurons lacking SV2 protein expression, meaning that SV2 proteins act in a post-docking priming step (Custer et al., 2006). Alterations of the RRP after SV2A depletion have been detected also in peripheral sympathetic neurons (Vogl et al., 2015).

### Synaptic Vesicle Glycoproteins 2 Interact With the Extracellular Matrix

SV2 proteins interact with components of the extracellular matrix. The study of Son et al. (2000) showed that purified SV2 proteins directly bind laminin-1 with high affinity. This interaction is likely to be activity-dependent, as it happens when SV2 proteins transiently become plasma membrane proteins after vesicular fusion. The capability of SV2 proteins to act as laminin receptors may be essential to the processes of axonal regeneration or the activity-dependent synaptic adhesion (Son et al., 2000).

### Synaptic Vesicle Glycoproteins 2 Mediate the Cellular Entrance of Neurotoxins

The interactions between SV2 proteins and neurotoxins have been thoroughly investigated. It has been demonstrated that SV2 proteins mediate the neuronal entrance of neurotoxins, in particular the botulinum neurotoxins (BoNTs). BoNTs are among the most dangerous known bacterial toxins, as they cause botulism, a condition characterized by the blockade of acetylcholine transmission and flaccid paralysis. The 7 main serotypes (BoNT/A-G) exploit the synaptic proteins to enter inside neurons; in particular, the serotypes BoNT/A (Dong et al., 2006), BoNT/D (Kroken et al., 2011; Peng et al., 2011), BoNT/E (Dong et al., 2008; Mahrhold et al., 2013) and BoNT/F (Fu et al., 2009; Rummel et al., 2009) use the SV2 luminal domain as a surface receptor and take advantage of the vesicle recycling mechanism to enter the cells. The role of SV2 proteins as receptors for botulinum neurotoxins was demonstrated initially for the BoNT/A serotype with neurons in which SV2 proteins were knocked-out and the uptake of neurotoxins was prevented. The uptake could be re-established by restoring SV2 expression (Dong et al., 2006).

For the serotype A, immunoelectron microscopy showed that the neurotoxin is internalized in the lumen of small synaptic vesicles, and not in other endosomal compartments, followed by a fast cytosolic translocation of the neurotoxin. Each small vesicle can contain one or two toxin molecules, indicating that SV2 proteins regulate the amount of toxin molecule present in a single vesicle (Colasante et al., 2013). It is noteworthy that neurotoxin subtypes that exploit SV2 proteins establish a very different interaction from the subtypes that bind to other synaptic proteins; for example, BoNT/A-SV2 interaction has very different properties compared to the interaction between BoNT/B and the protein synaptotagmin (Weisemann et al., 2016). Several studies revealed the specificity of neurotoxin subtypes for the different SV2 isoforms and the properties of interaction. As an example, the subtype BoNT/A1 interacts mainly with the isoforms SV2C, and SV2C glycosylation is fundamental to the toxin’s recognition of both the peptide moieties ad the SV2C-linked glycan (Yao et al., 2016). High resolution crystal structure revealed the specific interaction between the neurotoxin subtype BoNT/A2 and the luminal domain of SV2C (Gustafsson et al., 2018). It has been shown that SV2 proteins (particularly SV2A and SV2B isoforms) are also responsible for the neuronal entry of tetanus toxins that cause rigid paralysis (Yeh et al., 2010). Botulinum and tetanus neurotoxins share the same mechanism of entry into central neurons, by binding SV2 proteins during vesicular recycling (Blum et al., 2012).

### Other Functions of Synaptic Vesicle Glycoproteins 2

SV2 proteins are extremely multifaceted and possibly involved in more processes than those already described. Some of the additional hypothesized roles for SV2 proteins open promising research possibilities and are summarized as follows: The SV2A isoform is believed to have a mitochondrial localization and function. This hypothesis is suggested by the observed amelioration of mitochondrial dysfunctions typical of patients with Alzheimer’s disease upon administration of levetiracetam, the drug known to interact specifically with SV2A. This observation, together with the similarity between mitochondrial and vesicular membranes, led to the hypothesis of a mitochondrial role of SV2A, possibly as a fusion or fission factor (Stockburger et al., 2016). This finding could be extremely relevant to the study of pathological processes that involve mitochondrial dysfunctions.

SV2 proteins also are linked to the cellular energy balance by interaction with ATP. Yao and Bajjalieh (2008) reported that SV2A and SV2B bind to adenine-containing nucleotides, including ATP. The significance of this interaction is not clear, but it has been suggested that nucleotide binding somehow can regulate functions of SV2 proteins (Yao and Bajjalieh, 2008). Furthermore, interactions between SV2 isoforms and other synaptic proteins merit further investigations. Recently, cross-linking mass spectrometry studies uncovered an intricate network of links between the isoform SV2A and other synaptic vesicle proteins, such as synaptophysin and synaptobrevin-2 (Wittig et al., 2021). These interactions involve both the luminal and cytosolic domains of the proteins.

**FIGURE 1:**
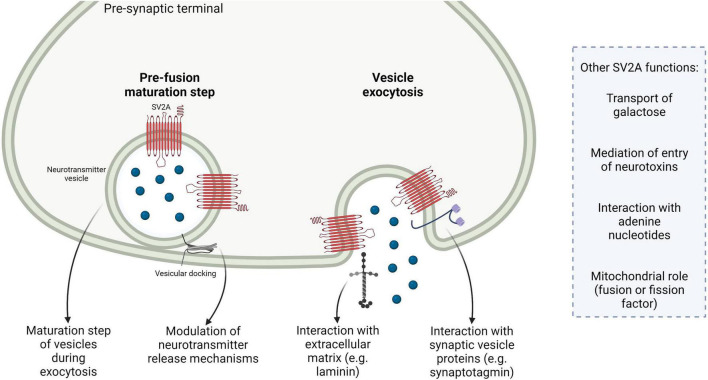
Summary of the main putative roles of the isoform synaptic vesicle glycoprotein 2A (SV2A). SV2A participates in a pre-fusion maturation step during vesicular exocytosis; SV2A is involved in neurotransmission *via* multiple mechanisms, e.g., by modulating the coupling between calcium entry and neurotransmitter release or by regulating machinery involved in neurotransmitter release; SV2A interacts with extracellular matrix components, e.g., laminin; SV2A interacts with other synaptic vesicle proteins, such as the calcium-sensor synaptotagmin. Other SV2A possible functions are reported, for example the transport of galactose, mediation of vesicular entry of neurotoxins, interaction with adenine nucleotides and mitochondrial localization and role as fusion or fission factor.

In summary, SV2 proteins seem mainly to have an important modulatory role in neurotransmission, especially by regulating a maturational step of vesicles during exocytosis and the coupling between calcium entry in the pre-synaptic terminal and release of neurotransmitter. The interaction with the calcium-sensor synaptotagmin is important. Other functions, especially the specific transport of galactose mediated by the isoform SV2A, the interaction with extracellular proteins like laminin, the interaction with adenine nucleotides and the possible mitochondrial localization and function are of interest and merit further investigations to fully understand SV2A protein roles in physiological and pathological conditions (Figure 1).

## Synaptic Vesicle Glycoprotein 2A and Levetiracetam

### The Anti-epileptic Drug Levetiracetam

Among the SV2 isoforms, SV2A has attracted particular attention due to the discovery that SV2A is the specific target of levetiracetam [(S)-α-ethyl-2-oxo-pyrrolidine acetamide], also known by the commercial name Keppra^®^. Levetiracetam is an atypical second-generation anti-epileptic drug, approved in 1999 by the Food and Drug Administration. The drug was discovered by random screening to have effects against different types of induced seizures, delineating a unique profile of action with features in common with different types of conventional anti-epileptic drugs (AEDs).

Levetiracetam has an interesting profile because of its broad spectrum of activity, high tolerability, and only few adverse effects; rodents treated with the drug showed only modest behavioral effects, such as a slight hyperactivity, even at high doses (Gower et al., 1992; Löscher and Hönack, 1993; Klitgaard et al., 1998). Along with the anticonvulsant activity, levetiracetam appears to exert antiepileptogenic effects. For example, it has been reported that levetiracetam is effective in preventing development and acquisition of pentylenetetrazole kindling (Ohno et al., 2010).

Levetiracetam is also characterized by long-term efficacy. A spontaneously epileptic rat model was used to test the efficacy of levetiracetam in comparison with conventional AEDs such as phenytoin, phenobarbital, valproate and carbamazepine. The results showed that levetiracetam is effective against seizures after single and repeated administration, with induction of long-lasting antiepileptic effects that are not reported with other AEDs (Ji-qun et al., 2005).

Epilepsy is associated with increased excitatory neuronal activity, and levetiracetam has been shown to prevent the hyperexcitability, exemplified by chronic treatment that fully prevents the development of hippocampal hyperexcitability in a model of pilocarpine-induced status epilepticus (Margineanu et al., 2008). In a rat neonatal model of hypoxia-induced seizures, levetiracetam has an anticonvulsant effect that renders long-term protection against behavioral seizures and neuronal injury (Talos et al., 2013).

### Mechanisms of Action of Levetiracetam

How levetiracetam exerts its anticonvulsant and antiepileptic effects upon specific binding to SV2A remains elusive. In different studies, researchers have tried to identify the means by which levetiracetam affects synaptic functions preventing excessive neuronal activation. Yang et al. (2007) proposed that levetiracetam directly interferes with pre-synaptic neurotransmitter release. Electrophysiological and cellular imaging techniques revealed that prolonged exposure to levetiracetam reduces the rate of release of pre-synaptic vesicles, which is consistent with the localization and the hypothesized functions of the target SV2A (Yang et al., 2007). Electrophysiological evidence suggested that levetiracetam acts as an inhibitor of neurotransmission by decreasing the pool of the readily releasable vesicles, and so possibly reducing neurotransmission (Meehan et al., 2011). In antral mucous cells of guinea pig, which contain SV2A, levetiracetam reduces calcium-mediated exocytosis by counteracting the functions of SV2A and affecting the priming of granules (Harada et al., 2013).

The mechanisms of action of levetiracetam are possibly more numerous, with effects as diverse as inhibition of endocytosis and enhancement of short-term depression (Li and Xue, 2018). The ability of levetiracetam to trigger short-term plasticity mechanisms is also of interest; over-active synapses undergo short-term depression process, known as supply rate depression, and levetiracetam appears to be able to enhance this process by shortening the induction time. The effect is not supported by SV2A knock-out neurons, once again consistent with the specific SV2A targeting of levetiracetam (García-Pérez et al., 2015). Along with the increase in short-term depression, levetiracetam reduces long-term potentiation. In a model of long-term potentiation induced in motor cortex by paired associative transcranial magnetic stimulation, the administration of levetiracetam reduced the long-term potentiation-like plasticity, a mechanism that possibly contributes to the antiepileptic action of the drug (Heidegger et al., 2010). In addition, there is general agreement that levetiracetam inhibits pre-synaptic voltage-activated calcium channels and reduces neuronal excitability through an intracellular pathway (Vogl et al., 2012).

Levetiracetam’s effects have been tested in a model of SV2A overexpression. The overexpression results in abnormal neurotransmission with decreased excitatory post-synaptic current amplitude and decreased synaptic depression, implying that an excessive presence of SV2A in the synapse is as aberrant as are low levels. Application of levetiracetam restores the normal synaptic transmission in SV2A-overexpressing terminals, and levetiracetam appears to be able to regulate SV2A localization in the synapses, rather than to influence its expression and turnover (Nowack et al., 2011).

As all synapses express SV2A ubiquitously, the logical consequence is that levetiracetam can produce its effects at both excitatory and inhibitory synaptic terminals. Meehan et al. (2012) demonstrated that levetiracetam also affects inhibitory neurotransmission, as it is able to reduce the release of GABA in a manner that qualitatively is similar to the reduction of the excitatory neurotransmission. The authors provided several explanations of how levetiracetam ameliorates an epileptic phenotype while acting on both excitatory and inhibitory systems. Among other reasons, the authors suggested that the dominance of excitatory hyperactivity in epilepsy makes the effects of levetiracetam preponderant in excitatory neurons. Alternatively, it is possible that levetiracetam would desynchronize GABA release, potentially slowing the inhibitory post-synaptic current time course and resulting in an overall increased inhibition (Meehan et al., 2012).

### Synaptic Vesicle Glycoprotein 2A Is the Specific Target of Levetiracetam

The first evidence for SV2A as a specific target of levetiracetam derives from the study of Lynch et al. (2004) who showed that the drug is unable to bind to brain membranes and purified synaptic vesicles from mice lacking SV2A expression, and the SV2B and SV2C isoforms do not support the binding (Lynch et al., 2004). Moreover, the partial reduction of SV2A expression in a heterozygous SV2A-deficient mouse model is sufficient to halve the anti-epileptic effects of levetiracetam (Kaminski et al., 2009). Gillard et al. (2006) compared the binding features of levetiracetam in post-mortem human brains and Chinese hamster ovary cells expressing human SV2A. The binding was saturable and reversible, and the binding kinetics matched a two-phase model (Gillard et al., 2006).

The ability of levetiracetam to interact with SV2A, a pre-synaptic protein, is particularly relevant as levetiracetam differs from traditional AEDs that usually target channels or post-synaptic receptors. It is unclear how levetiracetam improves the epileptic phenotype upon SV2A binding, and a number of levetiracetam derivatives that maintain SV2A selective targeting and eventually have higher pharmacological effects are under scrutiny. In particular, brivaracetam is the main levetiracetam analog, with significant effects on neuronal excitability possibly due to multiple ionic mechanisms (Hung et al., 2021).

Understanding how SV2A and its tracers bind is a major issue as exact knowledge of the dynamics of the interaction could lead to more accurate interpretation of neuroimaging results and new discoveries. SV2A PET tracers derive from the drug levetiracetam, from which they inherit the specific targeting of SV2A, but the mechanism of entry of levetiracetam into neurons is not completely clear, and different mechanisms could mean different possibilities for the derived tracers to find and interact with the target, leading to different results and conclusions in neuroimaging.

### Entry Mechanism of Levetiracetam

Not many studies have focused on the molecular interaction between SV2A and levetiracetam. The most accredited hypothesis was discussed by Meehan et al. (2011) according to whom levetiracetam is thought to use synaptic vesicles to enter neurons and bind to the luminal side of SV2A, exploiting the recycling and endocytosis of vesicles. The hypothesis is supported by electrophysiological evidence, showing that the increase of synaptic transmission by manipulation of spontaneous activity or application of stimulation protocols raises the pharmacological effects of levetiracetam. Onset and magnitude of action of levetiracetam seem to be higher at neuronal activity of higher frequency, consistent with the observation that more vesicles fuse and give the drug better access to its target. It is noteworthy that in the same work, the authors discussed a possible controversy: during high-frequency stimulations, some synapses present the “kiss-and-run” release mechanism, in which the vesicles do not undergo complete fusion with the membrane and are re-used multiple times. In this case, levetiracetam would have access only to a limited number of fusing vesicles and the pharmacological effects would be negligible. As discussed by the authors, an alternative entry mechanism of levetiracetam could be the direct crossing of the plasma membrane and interaction with the target SV2A (Meehan et al., 2011).

### Putative Binding Site of Levetiracetam on Synaptic Vesicle Glycoprotein 2A

The understanding of the exact binding site of levetiracetam and the derived SV2A tracers on SV2A is a major issue, as the point of interaction leads to different interpretations of the neuroimaging findings. A combination of theoretical and experimental approaches helped the identification of a series of amino acidic residues possibly involved in the racetams binding to SV2A, localized in transmembrane regions of the protein (Shi et al., 2011; Correa-Basurto et al., 2015).

Interestingly, protein tomography identified two possible configurations of SV2A, a funnel structure with a pore-like opening toward the cytoplasm and a V-shaped structure with a cleft-like opening toward the vesicular lumen. This two-configuration model is coherent with the hypothesized transporter function of SV2A and could help the localization of the binding site of levetiracetam (Lynch et al., 2008). In a later study (Lee et al., 2015), the two configurations of SV2A protein were further characterized, and the outward configuration (with the putative binding site exposed to the vesicular lumen) was considered a more probable description of levetiracetam’s interaction than the inward configuration (with the binding site exposed to the cytoplasm), consistent with the hypothesis of the vesicular entry of the drug suggested by Meehan et al. (2011). However, according to Lynch et al. (2008), the two configurations of SV2A appear to be present in samples treated with levetiracetam, suggesting that the drug fails to provoke large configuration changes of the protein upon its binding and, more importantly, that it may not require a specific SV2A configuration to bind. Taken together, the results do not provide a definitive answer to the question of the interaction between SV2A and levetiracetam, although they may favor the vesicular entry of the drug.

## Synaptic Vesicle Glycoprotein 2A and Positron Emission Tomography Imaging

### Synthesis and Validation of Synaptic Vesicle Glycoprotein 2A Positron Emission Tomography Tracers

The specificity of the interaction between levetiracetam and SV2A raised the possibility of exploiting this binding to label the SV2A protein in studies of changes of expression in health and disease. Since SV2A is a vesicular protein expressed in all the pre-synaptic terminals, it was thought to be a faithful indicator of the synaptic density. Altered SV2A density would then be related to changes of the density of synapses, and this is particularly relevant to the *in vivo* evaluation of physiological or pathological conditions that involve alterations of the density of synapses.

Currently, much effort is exerted to produce radiolabeled tracers of SV2A that can be applied to PET imaging. Among the noteworthy attempts are the generation of a first version of levetiracetam labeled with carbon-11 (Cai et al., 2014) and the later version labeled with the metastable radioisotope technetium 99, used in single photon emission computed tomography after intranasal administration (Rashed et al., 2018). Consequently, using the tridimensional pharmacophore model of levetiracetam and strict analogs, new compounds were designed with the same specificity and eventually higher affinity for SV2A than the drug itself, including UCB-A, UCB-H and UCB-J; these molecules are suitable for PET radiolabeling (Mercier et al., 2014).

Numerous studies were performed to synthetize, evaluate and validate radiolabeled UCB-A (Estrada et al., 2016) and UCB-H (Bretin et al., 2013, 2015; Warnock et al., 2014; Warnier et al., 2016; Becker et al., 2017; Serrano et al., 2019a,b). In a study with healthy human volunteers, [^18^F]UCB-H uptake appeared to be ubiquitous in cortical and subcortical gray matter areas, reflecting the pattern of distribution of SV2A and so proving the reliability of UCB-H as a SV2A marker (Bahri et al., 2017). Recently, the pharmacokinetics of [^18^F]UCB-H were re-evaluated in healthy primate brains, revealing good parameters but a lower specific signal compared to the more recently developed tracers (Goutal et al., 2021).

[^11^C]UCB-J has proven itself to have excellent properties for PET imaging, with high uptake, fast kinetics, and whole-body biodistribution in non-human primates. The uptake is particularly high in gray matter brain areas, which is consistent with SV2A expression (Nabulsi et al., 2016). Validation studies in humans and non-human primates showed the SV2A-specific binding of [^11^C]UCB-J and its favorable imaging properties; a first *in vivo* [^11^C]UCB-J PET study in epileptic patients demonstrated its ability to detect changes in “synaptic density” in a non-invasive way (Finnema et al., 2016). [^11^C]UCB-J also presents favorable kinetic features and good test-retest reproducibility of binding parameters (Finnema et al., 2018). A white matter region, the centrum semiovale, was accepted as an adequate reference region for [^11^C]UCB-J PET analyses of non-human primate and human brains (Koole et al., 2019; Mertens et al., 2020; Rossano et al., 2020). The [^11^C]UCB-J radiation dosimetry was estimated by whole-body imaging of human adolescents and adults, with detection of residence times in different organs, resulting in higher values for brain and liver (Bini et al., 2020). A large human cohort study revealed that imaging for 60- to 90- min is sufficient for the precise quantification of [^11^C]UCB-J specific binding (Naganawa et al., 2021a). Preclinical studies have been performed to evaluate [^11^C]UCB-J PET in different animal models, including mice (Bertoglio et al., 2020), rats (Thomsen et al., 2021b) and minipigs (Thomsen et al., 2020).

The synthesis of improved SV2A PET markers is a rapidly developing field, and efforts are continuously made to produce the optimal SV2A radioligand. The improvement of synthesis and uptake evaluations are ongoing processes (Rokka et al., 2019; Milicevic Sephton et al., 2020). The results of [^11^C]UCB-J PET are promising, but its utilization is limited because of the short 20-min half-life of carbon-11. Thus, a [^18^F]-labeled version of UCB-J, with a half-life of 110 min, was synthetized and evaluated in PET studies (Li et al., 2019b). Several [^18^F]-labeled candidates have now been evaluated (Patel et al., 2020), especially UCB-J derivatives including [^18^F]MNI-1126 (Constantinescu et al., 2019), also named [^18^F]-SDM-8 and described by Li et al. (2019a) and then referred as [^18^F]-SynVesT-1 (Li et al., 2021; Naganawa et al., 2021b). Other recently developed compounds are [^18^F]-SynVesT-2 (Cai et al., 2020) and [^18^F]-SDM-16 (Zheng et al., 2021).

In order to assess tracer specificity, Nabulsi et al. (2016) performed PET studies in non-human primates in the presence of high doses of unlabeled UCB-J or levetiracetam, which resulted in a reduction of [^11^C]UCB-J binding compared to baseline, thus confirming the competition between the tracer and levetiracetam for the same binding sites. These findings were confirmed in humans by Finnema et al. (2016). The displacement of [^11^C]UCB-J later was evaluated after the administration of therapeutically relevant doses of levetiracetam and brivaracetam in healthy volunteers that resulted in a blockade of [^11^C]UCB-J binding by both drugs. Brivaracetam had a higher brain penetration and achieved high SV2A occupancy faster than levetiracetam, suggesting its higher affinity for the molecular target SV2A that may be therapeutically relevant (Finnema et al., 2019).

At the current state-of-art, SV2A PET imaging is a powerful tool used to evaluate potential changes in synaptic SV2A density in pathological and physiological contexts, as reported below (Table 2).

**TABLE 2:** Synaptic vesicle glycoprotein 2A (SV2A) density changes detected with positron emission tomography (PET) in different conditions.

Condition	Model	References
Alzheimer’s disease	Human	Chen et al. (2018, 2021), Bastin et al. (2020), Mecca et al. (2020, 2021, 2022), Lu et al. (2021), O’Dell et al. (2021)
	Mouse	Toyonaga et al. (2019), Sadasivam et al. (2021), Xiong et al. (2021)
Parkinson’s disease	Human	Delva et al. (2020), Matuskey et al. (2020), Wilson et al. (2020)
	Mouse	Xiong et al. (2021)
	Rat	Thomsen et al. (2021a,b), Raval et al. (2021a)
Dementia with Lewy body	Human	Nicastro et al. (2020), Andersen et al. (2021)
Frontotemporal dementia	Human	Salmon et al. (2021)
Huntington’s disease	Human	Delva et al. (2021)
	Mouse	Bertoglio et al. (2021)
	Rat	Thomsen et al. (2021b)
Progressive supranuclear palsy, corticobasal degeneration	Human	Holland et al. (2020)
Epilepsy	Human	Finnema et al. (2020)
	Rat	Serrano et al. (2020)
Schizophrenia	Human	Onwordi et al. (2020, 2021), Radhakrishnan et al. (2021)
Depression and post-traumatic stress disorder	Human Human and non-human primates	Holmes et al. (2019), Holmes et al. (2022)
Substance use disorder	Human	Angarita et al. (2021), D’Souza et al. (2021)
Normal aging	Human	Michiels et al. (2021a)
HIV infection	Human	Weiss et al. (2021)
Ischemic stroke	Human	Michiels et al. (2021b)
Obesity	Human	Asch et al. (2021)

### Synaptic Vesicle Glycoprotein 2A Positron Emission Tomography Imaging in Neurodegenerative Disorders

#### Alzheimer’s Disease

SV2A PET is useful in studies of progressive pathologies, such as neurodegeneration, and in the evaluation of the effects of drugs and treatments. The decline in synaptic density is a pathological hallmark of neurodegenerative diseases. In Alzheimer’s disease patients, PET imaging with different SV2A tracers revealed a decreased binding in several cortical areas, thalamus (Bastin et al., 2020) and hippocampus (Chen et al., 2018; Bastin et al., 2020). Another study showed reduced binding in the medial temporal cortex and neocortical areas (Mecca et al., 2020). Furthermore, in the medial temporal lobe, similar reductions were observed comparing [^11^C]UCB-J binding and [^18^F]-fluoro-deoxy-glucose ([^18^F]FDG) uptake as a measure of neuronal metabolic activity (Chen et al., 2021). Interestingly, contrary to these findings, a post-mortem study with [^3^H]UCB-J autoradiography revealed no differences of binding in the frontal cortex of Alzheimer’s patients compared to control subjects (Metaxas et al., 2019).

The binding of [^11^C]UCB-J was also compared with fibrillar Aβ deposition in patients with early Alzheimer’s disease, with amnestic mild cognitive impairment or mild dementia. Aβ deposition was more strongly inversely correlated with SV2A density in hippocampi of patients with amnestic impairment than in patients with dementia, although the authors suggested that Aβ deposition could reach a plateau phase in which there is no longer correlation with synaptic loss (O’Dell et al., 2021). Mecca et al. (2021) found a reduction in SV2A density in the hippocampus of early Alzheimer’s disease patients measured with [^11^C]UCB-J PET, inversely associated with tau deposition in the entorhinal cortex and probably due to the degeneration of entorhinal cortex neurons that project to the hippocampus in the early phase of the disease (Mecca et al., 2021). In a more recent study, [^11^C]UCB-J distribution volume ratio was found to be a good predictor of global cognitive performance in Alzheimer’s disease patients analyzed with an extensive neuropsychological test battery, allowing the correlation between synaptic density and cognitive dysfunction (Mecca et al., 2022).

Synaptic vesicle glycoprotein 2A (SV2A) PET may aid in the evaluation of drug effects on synaptic density. In a mouse model of Alzheimer’s disease, [^11^C]UCB-J binding indicated synaptic loss in the hippocampus that was then restored after the administration of saracatinib, an experimental drug for the treatment of Alzheimer’s disease (Toyonaga et al., 2019).

Continuous efforts aim to improve the analyses and interpretation of SV2A PET results in Alzheimer’s disease. New studies apply partial volume correction algorithms to verify the true SV2A density changes in the patients (Lu et al., 2021) and to make use of the most recent and best-performing tracers in animal models of the disease (Sadasivam et al., 2021). The group of Xiong et al. (2021) created two transgenic mouse models for Alzheimer’s and Parkinson’s disease and performed [^11^C]UCB-J PET to investigate synaptic loss in different brain areas (Xiong et al., 2021).

#### Parkinson’s Disease

The first evidence of synaptic loss in living patients with Parkinson’s disease obtained with [^11^C]UCB-J PET was found in brain areas involved in the pathogenesis of the disease, including the substantia nigra, red nucleus, locus coeruleus, and relevant cortical regions (Matuskey et al., 2020). Another study found a significant decrease in [^11^C]UCB-J binding in the substantia nigra of patients with early Parkinson’s disease, compared to healthy control subjects (Delva et al., 2020).

Wilson et al. (2020) investigated possible correlations between mitochondrial, endoplasmic reticulum and synaptic dysfunctions in early drug-naïve Parkinson’s patients using PET tracers for mitochondrial complex 1, sigma 1 receptor and SV2A, with [^18^F]-BCPP-EF, [^11^C]-SA-4503, and [^11^C]UCB-J, respectively (Wilson et al., 2020). The kinetics of these three PET markers had been evaluated in a previous work (Mansur et al., 2020) where [^11^C]UCB-J, used for SV2A detection, showed a significant decrease in volume of distribution in the caudate, putamen, thalamus, brain stem, dorsal raphe and cortical areas of patients compared to healthy controls. Interestingly, no significant changes were detected in the volume of distribution of [^18^F]-BCPP-EF and [^11^C]-SA-4503 in these regions (Wilson et al., 2020).

The tracer [^11^C]UCB-J was further tested by visualization *in vivo* as a marker of synaptic SV2A density loss in rats with unilateral striatal lesions using 6-hydroxydopamine (6-OHDA) to model toxin-induced Parkinson’s disease (Thomsen et al., 2021b) or preformed α-synuclein fibrils to model progressive Parkinson’s disease (Thomsen et al., 2021a). In both studies, small (6%) reductions of SV2A binding were observed in the ipsilateral side of the brain; these small changes were consistent with views that dopamine projections from the substantia nigra constitute only 10% of terminals in the striatum (Groves et al., 1994; Uchigashima et al., 2016). The same team did [^3^H]UCB-J autoradiography of brain sections from an early model of Parkinson’s disease induced by unilateral intranigral injection of recombinant adeno-associated viral vectors expressing the human α-synuclein protein, but did not find changes of SV2A density in the striatum or substantia nigra in this early model, despite the reduction of dopaminergic pre-synaptic terminal markers including the dopamine transporter and the vesicular monoamine transporter 2 (Stokholm et al., 2021). A transgenic mouse model of Parkinson’s disease was developed and assessed with [^11^C]UCB-J PET in the work mentioned above by Xiong et al. (2021). Raval et al. (2021a) used the 6-OHDA rat model to investigate correlations between changes in SV2A density and neuronal metabolism with [^11^C]UCB-J and [^18^F]FDG PET, finding a similar decrease of both [^11^C]UCB-J and [^18^F]FDG signals in the basal ganglia of the lesioned hemisphere (Raval et al., 2021a).

SV2A imaging may be useful as a tool to study the effects of treatments on alleged synaptic loss associated with neurodegenerative disorders. For example, 6-OHDA-injected parkinsonian rats subjected to treadmill exercise were found to have restored striatal and nigral [^3^H]UCB-J binding by *in vitro* autoradiography (Binda et al., 2021).

#### Dementia With Lewy Bodies

[^11^C]UCB-J binding was used to verify changes in synaptic density in patients with dementia with Lewy bodies, showing that patients display reduced signal especially in parietal and occipital regions (Nicastro et al., 2020). A widespread reduction of cortical synaptic density was also confirmed by Andersen et al. (2021) in patients with Parkinson’s disease dementia and dementia with Lewy bodies.

#### Frontotemporal Dementia

[^18^F]UCB-H uptake was assessed in patients with a behavioral variant of frontotemporal dementia, showing a trend toward a decrease in the distribution volume of the tracer in the right parahippocampal region compared to healthy subjects. Comparisons between patients with the behavioral variant of frontotemporal dementia and data from a group of patients with Alzheimer’s disease did not result in significant differences (Salmon et al., 2021).

#### Huntington’s Disease

Loss of synapses was reported with SV2A microPET in a heterozygous knock-in Q175DN mouse model of Huntington’s disease. The values were confirmed with [^3^H]UCB-J autoradiography and SV2A immunofluorescence (Bertoglio et al., 2021). SV2A microPET and autoradiography also revealed dose-dependent reductions of striatal SV2A binding in response to quinolinic acid injected in rat to induce an acute lesion model of Huntington’s disease (Thomsen et al., 2021b). A study of patients with premanifest and early Huntington’s disease revealed reduced [^11^C]UCB-J binding in putamen, caudate, pallidum, cerebellum, and parietal, temporal, and frontal cortices of the patients. Comparative analyses with [^18^F]-FDG revealed reduced metabolism only in putamen and caudate (Delva et al., 2021).

#### Other Neurodegenerative Pathologies

A decrease of synaptic SV2A density was assessed in a study of progressive supranuclear palsy (Richardson’s syndrome) and in amyloid-negative corticobasal syndrome. [^11^C]UCB-J detection revealed greatly reduced binding in frontal, temporal, parietal, and occipital lobes, cingulate, hippocampus, insula, amygdala and subcortical areas in both pathologies (Holland et al., 2020).

### Synaptic Vesicle Glycoprotein 2A Positron Emission Tomography Imaging in Other Neurological Disorders and Neuropsychiatric Disorders

#### Epilepsy

The synthesis of SV2A tracers from the pharmacophore of the anti-epileptic drug levetiracetam makes the application of SV2A PET highly relevant to studies of epilepsy. The expression of SV2A in epilepsy displays a complex and variable pattern during the progression of the pathology. Thus, [^18^F]UCB-H PET detected SV2A variations in specific cases of kainic acid-induced temporal lobe epilepsy during the distinct phases of the pathological progression (early, late, transition and chronic). The analysis showed a progressive increase of [^18^F]UCB-H binding in healthy animals, and a smaller increase in epileptic animals along the phases. In the last two phases, [^18^F]UCB-H binding was significantly reduced in epileptic animals compared to control subjects in several brain areas (Serrano et al., 2020). The reduction of SV2A expression in temporal lobe epilepsy was seen also in a study of [^11^C]UCB-J PET in epileptic patients. In particular, reductions of SV2A density in the epileptogenic (ipsilateral) medial temporal lobe were detected in comparison with the contralateral lobe, consistent with the putative seizure onset zone, making SV2A imaging useful in the pre-surgical evaluation of patients (Finnema et al., 2020).

#### Schizophrenia

Investigations of neuropsychiatric disorders also adopted SV2A PET imaging. Onwordi et al. (2020) reported reduced binding of [^11^C]UCB-J in frontal and anterior cingulate cortices of patients with schizophrenia compared to control subjects. The administration of antipsychotic drugs failed to change SV2A levels in frontal cortex of healthy rats determined by [^3^H]UCB-J autoradiography and immunohistochemistry, implying that antipsychotic medications have no effect on SV2A expression (Onwordi et al., 2020). A later study reported that neither the chronic exposure to antipsychotic drugs such as haloperidol and olanzapine, nor the administration of lithium had effects on SV2A levels, measured with immunochemistry (Halff et al., 2021).

Radhakrishnan et al. (2021) also found a reduction of [^11^C]UCB-J binding in patients with schizophrenia, especially in frontal, anterior cingulate, occipital, parietal and temporal cortices and hippocampus compared with healthy controls (Radhakrishnan et al., 2021).

Glutamatergic dysfunction is an important factor in schizophrenia onset, and SV2A density and glutamate levels in anterior cingulate cortex and hippocampus have been measured in patients with schizophrenia compared to healthy volunteers with [^11^C]UCB-J PET. Interestingly, no significant relationship was reported between [^11^C]UCB-J binding and glutamate levels in the patients, while a positive correlation was detected in healthy control subjects (Onwordi et al., 2021).

#### Depression and Post-traumatic Stress Disorder

The group of Holmes et al. (2019) tested individuals with major depressive disorder, post-traumatic stress disorder or both, with [^11^C]UCB-J PET, revealing an inverse correlation between the severity of depressive symptoms and SV2A density measure in the dorsolateral prefrontal and anterior cingulate cortices, and hippocampus. In the same study, the authors discovered aberrant network connectivity by magnetic resonance imaging of patients compared to healthy individuals, including in particular lower whole-brain resting functional connectivity in the dorsolateral prefrontal cortex (Holmes et al., 2019).

The psychedelic substance psilocybin appears to have antidepressant effects. In a study with [^3^H]UCB-J autoradiography, acute administration of psilocybin persistently raised the SV2A density measure in prefrontal cortex and hippocampus in pig brain. Psilocybin also induced an acute decrease in serotonin receptor levels in the same areas, which did not persist 7 days after the treatment. These findings could contribute to the explanation of how psilocybin produces its antidepressant action (Raval et al., 2021b).

In a recent study of Holmes et al., [^11^C]UCB-J PET scans were performed to search for possible alteration of SV2A density in response to ketamine, which is known to have antidepressant effects. The study was performed in patients with major depressive disorder or post-traumatic stress disorder, and in healthy control subjects. Despite the evident reduction of the depressive symptoms, no significant differences of [^11^C]UCB-J binding were detected in patient or control groups 24 h after the administration of a single dose of ketamine, in any analyzed brain areas (dorsolateral prefrontal cortex, anterior cingulate cortex, hippocampus). The authors also completed a *post hoc* study, showing that patients with lower SV2A density at baseline may exhibit an increased SV2A density 24 h after ketamine administration, coherent with the reduction in severity of depression (Holmes et al., 2022).

#### Substance Use Disorder

Recent studies focused on the detection of synaptic loss with SV2A PET associated with substances of abuse. In cannabis consuming subjects, PET analyses demonstrated a significant lowering of [^11^C]UCB-J binding in the hippocampus (D’Souza et al., 2021). A [^11^C]UCB-J PET study with humans affected by cocaine use disorder revealed a decrease in SV2A density in the prefrontal cortex, demonstrating the regional alteration of synapses induced by cocaine (Angarita et al., 2021).

### Synaptic Vesicle Glycoprotein 2A Positron Emission Tomography in Other Conditions

#### Normal Aging

[^11^C]UCB-J PET has been used to test if age and sex are factors associated with abnormal synaptic density in the human brain. The imaging results suggested that healthy aging is not correlated with cortical synaptic density loss, and no differences of synaptic SV2A binding were found between males and females (Michiels et al., 2021a).

#### Human Immunodeficiency Virus Infection

Recently, SV2A imaging was also applied to patients with HIV infection, as synaptic loss is a common hallmark of this condition. SV2A density was found to be reduced in the fronto-striatal-thalamic regions and other cortical areas of patients compared to control subjects (Weiss et al., 2021).

#### Ischemic Stroke

SV2A PET was used to evaluate changes of synaptic density in damaged brain tissue after ischemic stroke, resulting in a decrease of SV2A density in the lesioned area compared to healthy controls and in a lower [^11^C]UCB-J signal in the non-lesioned regions of the affected hemisphere compared to the unaffected one (Michiels et al., 2021b).

#### Obesity

A possible correlation between synaptic density and body mass index was tested in the study by Asch et al. (2021). Lower synaptic density was detected in overweight compared to normal weight participants; moreover, synaptic density was negatively correlated with body mass index especially in individuals with diagnosed stress-related psychiatric disorders, compared to mentally healthy controls (Asch et al., 2021).

## Synaptic Vesicle Glycoprotein 2A: Some Points of Reflection

### Synaptic Vesicle Glycoprotein 2A-Tracer Interactions and Relevance in Neuroimaging

SV2A imaging is a field of increasing interest in neuroscience, as it allows the *in vivo* evaluation of alterations interpreted as changes in synaptic density. However, there are some aspects of the SV2A molecule, and the use of SV2A markers, that have not been completely clarified by the literature, which may suggest the need for new experimental approaches aimed to more deeply understand the role of SV2A in neuroimaging.

Currently, it is assumed that levetiracetam, and possibly the derived SV2A tracers, all bind to the luminal side of SV2A, and it has been suggested that they could access their molecular target through the vesicle recycling and endocytosis process (Meehan et al., 2011). This is particularly relevant to the explanation of how the SV2A tracers enter the neurons and it would have important consequences for the interpretation of neuroimaging outcomes. For example, the possibility of a vesicular uptake of levetiracetam/SV2A tracers would signify that only vesicles that undergo recycling are labeled, thus representing only a subset of the entire vesicular content of the terminal. However, there are several critical shortcomings of this possibility. The synaptic vesicles belong to different functional pools, defined as readily releasable, recycling and reserve pools. The vesicles of the recycling pool maintain the release in conditions of physiological stimulation, and they represent the 5–20% of the entire vesicular content of the terminal (Rizzoli and Betz, 2005). Hence, if only recycling vesicles were labeled, the number of accessible targets for SV2A tracers would be too low and the radioactive signal probably too weak to efficiently allow detection of changed binding. A study by Smart et al. (2021) showed that [^11^C]UCB-J signal in fact remained unchanged in visual cortex of healthy volunteers during a visual stimulation task, despite the evidence of increased regional blood flow and thus tracer delivery, suggesting that SV2A is an unlikely indicator of vesicular dynamics and the recycling process, because the tracer signal would be expected to rise upon this stimulation in the cortical areas.

An alternative possibility, already mentioned in the work by Meehan et al. (2011) is the suggestion that the tracers cross the plasma membrane and the vesicular membrane to reach the target, thus potentially labeling the entire vesicular content of the terminal. This would be possible, as suggested by the biophysical features of SV2A tracers (e.g., lipophilicity and low molecular weight). However, even in this scenario, some mechanisms must be clarified, such as the accessibility of the tracer to the target. As the exact binding site of SV2A tracer on the luminal side of the protein is still uncertain, it would be interesting, for example, to precisely identify it and to verify if the two hypothesized conformational states of SV2A (Lynch et al., 2008; Lee et al., 2015) both equally enable the accessibility to the binding site, and, if not, to determine how SV2A conformation varies among the functionally different pools of vesicles in relation to vesicular dynamics. It is also important to ascertain whether all the SV2A copies in a single vesicle are labeled by the tracers, or only a subset.

**FIGURE 2:**
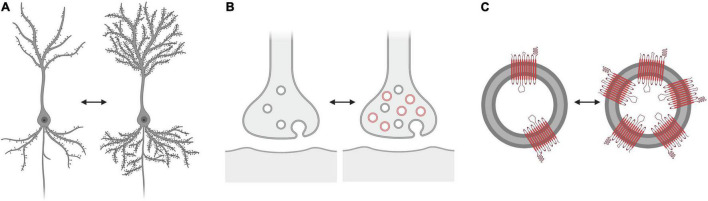
What does “altered SV2A density” represent? Three possibilities: **(A)** Altered synaptic vesicle glycoprotein 2A (SV2A) tracer binding could be associated with changes in “synaptic density” (exemplified as a change in the number of dendritic spines). **(B)** Altered SV2A tracer binding could be associated with changes in the number of vesicles in the pre-synaptic terminal, according to the functional state of the synapse, and so with functional properties of the synapses rather than their density. **(C)** Altered SV2A tracer binding could be associated with changes in the number of SV2A copies in the vesicles. The number of SV2A copies reached by the tracers and thus labeled may also vary. The alternative scenarios **(B)** and **(C)** do not necessarily implicate a change in the overall synaptic density.

Another noteworthy point is the meaning of “changed SV2A density.” The common interpretation is that a change of SV2A density is indicative of a change of the synaptic density, but it is important to consider possible alternatives, such as the variability of the number of vesicles related to the functional state of the synapse, and so of the presence of SV2A in a synaptic terminal according to its activity. It is also unclear if and how SV2A copy numbers may vary inside the same vesicles, with possible implications for imaging results (Figure 2). Future research may try to solve these issues, to figure out how the SV2A signal may be altered in relation to variations of vesicular trafficking, and whether these variations are related to changes of synaptic density. It remains to be clarified to what extent the SV2A density is related to the synaptic density, and how the SV2A protein is capable of representing the synapse as a whole. These queries aim to solicit further investigation of the SV2A protein and, ultimately, to understand what information is actually reported in neuroimaging studies.

### Synaptic Vesicle Glycoprotein 2A Is Preferentially Associated With GABAergic Terminals

SV2A is expressed in all synaptic terminals; however, recent studies reported that SV2A expression can be differentially associated with different types of synaptic terminals in some brain structures. Vanoye-Carlo and Gómez-Lira (2019) analyzed the expression of SV2A during the post-natal development of the rat hippocampus, using immunofluorescence to identify the distribution of SV2A and its co-localization either with the vesicular GABA or the glutamate transporters. The authors observed a preferential expression of SV2A in GABAergic terminals in the principal cells (granular and pyramidal) layers, while SV2A was mostly associated with glutamatergic terminals in hilus and stratum lucidum (Vanoye-Carlo and Gómez-Lira, 2019). Differential expression of SV2A in excitatory and inhibitory terminals was also assessed in the olfactory bulb, cortex, hippocampus and cerebellum of the adult rat brain. Immunofluorescence assays revealed SV2A and vesicular GABA transporter co-localization surrounding the principal cells of most of the layers of the analyzed structures, reinforcing a relationship between SV2A and the GABAergic system (Mendoza-Torreblanca et al., 2019).

Knowing how SV2A density is distributed in different synaptic terminal types, in different brain structures and in different conditions will help evaluate not only changes in “synaptic density,” but also the kind of neurotransmission affected in a particular condition, with relevant consequences for pathology studies. In the study by Contreras-García et al. (2021), the authors analyzed SV2A distribution and SV2A co-localization with vesicular glutamate and GABA transporters (VGLUT and VGAT, respectively) in different areas of the hippocampus of rats in an early state of pilocarpine-induced epilepsy. Epileptic rats were divided in two groups, saline-treated and levetiracetam-treated, and the latter group further divided in responders and non-responders to levetiracetam action based on the detected drug effects on seizures. SV2A total expression was unchanged among the experimental groups, however, the authors could observe a significant association of SV2A with inhibitory terminals, as principal and synaptic hippocampal layers showed high percentages of GABAergic terminals co-expressing SV2A, while lower percentages were observed in the glutamatergic terminals. Furthermore, SV2A-VGLUT co-localization was decreased in different hippocampal strata of non-responder compared to responder animals, suggesting an importance of SV2A expression in glutamatergic terminals for the responsiveness to levetiracetam. In contrast, no differences were detected in SV2A-VGAT co-localization, underlining the possibility that the inhibitory system maintains normal functionality in the early state of this epilepsy model (Contreras-García et al., 2021). Overall, the importance of SV2A in excitatory and inhibitory neurotransmission remains to be clarified.

In addition to the diverse expression in GABAergic and glutamatergic synapses, SV2A has been shown to contribute differentially to the synaptic vesicle recycling process in excitatory and inhibitory terminals. A recent study (Bae et al., 2020) demonstrated that SV2A is responsible for a 75% slower synaptic vesicle endocytosis rate in inhibitory compared to excitatory synapses of rat hippocampus. The difference probably was due to the differential expression of the protein, and the authors confirmed the higher expression of SV2A in inhibitory compared to excitatory terminals. Moreover, the overexpression of SV2A in excitatory neurons resulted in slower synaptic vesicle endocytosis kinetics, suggesting that the efficiency of the molecular process possibly is controlled by the amount of SV2A in the terminal. In the same work of Bae et al. (2020), the authors also demonstrated that SV2A seems to interact with synaptotagmin-I predominantly in inhibitory terminals, suggesting a role of SV2A as coordinator of synaptotagmin-I-mediated synaptic vesicle trafficking mostly in inhibitory neurons.

The differential participation of SV2A in different kinds of neurotransmission is poorly understood, and further studies are needed. Future research would likely improve the understanding of the differential expression of SV2A in different terminals and would clarify the apparent stronger SV2A association with GABAergic neurotransmission in particular brain structures and conditions.

### Synaptic Vesicle Glycoprotein 2A Participation in Excitation-Inhibition Balance

SV2A is essential to the maintenance of normal neurotransmission, and altered SV2A expression can affect the balance between excitation and inhibition, as it happens in the epileptic phenotype. Several studies examined how SV2A impacts the excitation-inhibition balance in neuronal circuits, especially in light of the stronger association between SV2A and GABAergic terminals. A mouse model lacking SV2A expression was created to investigate changes of GABAergic and glutamatergic transmissions in hippocampal neurons. Electrophysiological studies detected a reduction of spontaneous inhibitory post-synaptic currents, and an increase in spontaneous excitatory post-synaptic currents, indicating that GABAergic neurotransmission is affected by SV2A deficiency, and the excitation-inhibition balance is altered. Remarkably, both inhibitory and excitatory miniature post-synaptic currents were unaltered, implying that SV2A has no effect on activity-independent neurotransmitter release (Venkatesan et al., 2012).

### Synaptic Vesicle Glycoprotein 2A Expression in Epilepsy

Epilepsy demonstrates the intricate dynamics of SV2A expression, since it derives from a disruption of the excitation-inhibition balance in which SV2A seems to play a role. SV2A levels change during the development of the pathology and vary according to a complex pattern of different pathological stages that would hinder the interpretation of any change detected of SV2A. Generally, a decreased SV2A level (Feng et al., 2009; Kaminski et al., 2009; van Vliet et al., 2009; Hanaya et al., 2012; Shi et al., 2015; Menten-Dedoyart et al., 2016) or mutations of the *sv2a* gene (Douaud et al., 2011; Serajee and Huq, 2015; Harper et al., 2020) are associated with an epileptic phenotype. Hippocampal low frequency stimulation contributes to the rescue of SV2A expression as it ameliorates the epileptic phenotype in pharmacoresistant epileptic rats (Wang et al., 2014).

In 2016, a new rat model with a *sv2a* missense mutation was generated (Tokudome et al., 2016a,b). Although the basal GABA release in hippocampus and amygdala and the global SV2A expression level in inhibitory neurons remained unaffected, the authors observed reduced activity-induced GABAergic (but not glutamatergic) release and higher susceptibility of the animals to kindling-induced epileptogenesis, again suggesting the complicated relationship between SV2A and GABAergic transmission in epilepsy.

In a mouse pentylenetetrazole-kindled model with induced epileptic seizures, Ohno et al. (2009) demonstrated increased SV2A expression in GABAergic interneurons of the hilar region of dentate gyrus, probably as counteraction to the excessive excitatory activity that is typical of epilepsy. SV2A expression was unaltered in other hippocampal regions and in other brain areas, suggesting a region-specific protein up-regulation (Ohno et al., 2009). Furthermore, SV2A shows temporal changes of expression and patterns of association with GABAergic or glutamatergic systems in the hippocampus during the progression of epileptogenic pathology. Contreras-García et al. (2018) studied SV2A expression in a lithium-pilocarpine-induced temporal lobe epilepsy rat model, focusing on status epilepticus, early epilepsy and late epilepsy. After status epilepticus, the authors reported a region-specific increase of the SV2A level in principal cells layers, mainly associated with GABAergic terminals. SV2A levels were reduced to the baseline in early epilepsy; in late epilepsy, SV2A labeling was increased in stratum lacunosum-moleculare, and inner molecular and hilar layers, without a clear association with GABAergic or glutamatergic transmission. These findings indicate that SV2A density varies intriguingly during the evolution of epilepsy (Contreras-García et al., 2018). Similar studies are fundamentally important to identify when SV2A levels are altered in other brain structures and during progressions of other pathologies, especially of those that are studied with SV2A imaging, as the complicated expression patterns can confound the understanding of the imaging results.

## Conclusion

In this work, we summarized the current literature of SV2 proteins, focusing in particular on the isoform SV2A that has acquired increasing importance in the neuroimaging field in recent years. We reviewed the hypothetical functions of SV2 proteins, pointing out both accepted and hypothesized functions. We next reported the peculiarities of SV2A which is used as a candidate marker of synaptic density because of its ubiquitous expression throughout the brain and its specific binding by the anti-epileptic drug levetiracetam. Levetiracetam pharmacophore has been exploited to design specific SV2A radiolabeled tracers that currently are intensively used in neuroimaging, especially in PET. Despite the broad use of SV2A tracers, there are some unclear points about the utilization of the protein as a synaptic density marker. For example, the accessibility of the tracers to the molecular target should be further investigated, because it could have different meanings in neuroimaging, depending on whether the tracers are able to label all the targets in the synaptic terminal or only a subset of them. Moreover, SV2A expression seems to be preferentially associated with the GABAergic rather than the glutamatergic transmission, an observation that could help associating changes of SV2A distribution with alterations of a specific neurotransmission type in a particular condition. Finally, we reported that SV2A expression seems to be regulated in a complicated way, with very dynamic changes in time and space during the progression of epilepsy. The study of how the expression of SV2A varies temporally and regionally in epilepsy and other pathological contexts will improve our understanding of the pathological mechanisms, potentially pointing out new roles of SV2A, and helping in the evaluation of the SV2A imaging outcomes. In this way, SV2A would not just be a marker of a generalized synaptic density measure, but the distribution pattern and its alterations would provide an overall framework in a pathological or physiological context, from which it would be possible to extrapolate meaningful information.

## Author’s Note

In recent years, the development of radiolabeled tracers specific for the synaptic vesicle glycoprotein 2A (SV2A) significantly advanced neuroimaging with positron emission tomography (PET). SV2A is a ubiquitous transmembrane protein of synaptic vesicles now used as an index or measure of a variable known as “synaptic density.” The measure is held to allow *in vivo* evaluation of synaptic density under physiological or pathological circumstances. Despite the common use of SV2A tracers and the resulting evidence of changes to the measure, specific questions remain unanswered: What are the true biological roles of SV2A? What are the entry mechanisms of SV2A tracers, and where do they bind, e.g., to all vesicles in the pre-synaptic terminal, or selectively to a subset of vesicles? How do SV2A expression patterns vary in different contexts and brain structures? Here we discuss the use of SV2A as a general marker of the variable known as synaptic density, and we bring to light topics for further research with the ultimate aim of improving interpretation of the SV2A imaging outcomes in health and disease.

## Author Contributions

RR, SA, SB, AG, and AL: conceptualization. RR and AL: funding acquisition. RR: first draft. SA, SB, AG, and AL: revision. All authors approved the final version.

## Conflict of Interest

The authors declare that the research was conducted in the absence of any commercial or financial relationships that could be construed as a potential conflict of interest.

## Publisher’s Note

All claims expressed in this article are solely those of the authors and do not necessarily represent those of their affiliated organizations, or those of the publisher, the editors and the reviewers. Any product that may be evaluated in this article, or claim that may be made by its manufacturer, is not guaranteed or endorsed by the publisher.

## References

[B1] Andersen K. B. Hansen A. K. Damholdt M. F. Horsager J. Skjaerbaek C. Gottrup H. (2021). Reduced synaptic density in patients with lewy body dementia: an [(11) C]UCB-J PET imaging study. *Mov. Disord.* 36 2057–2065. 10.1002/mds.28617 33899255

[B2] Angarita G. A. Worhunsky P. D. Naganawa M. Toyonaga T. Nabulsi N. B. Li C. R. (2021). Lower prefrontal cortical synaptic vesicle binding in cocaine use disorder: an exploratory (11) C-UCB-J positron emission tomography study in humans. *Addict. Biol.* 27:e13123. 10.1111/adb.13123 34852401 PMC8891080

[B3] Asch R. H. Holmes S. E. Jastreboff A. M. Potenza M. N. Baldassarri S. R. Carson R. E. (2021). Lower synaptic density is associated with psychiatric and cognitive alterations in obesity. *Neuropsychopharmacology* 47 543–552. 10.1038/s41386-021-01111-5 34294874 PMC8674236

[B4] Bae J. R. Lee W. Jo Y. O. Han S. Koh S. Song W. K. (2020). Distinct synaptic vesicle recycling in inhibitory nerve terminals is coordinated by SV2A. *Prog. Neurobiol.* 194:101879. 10.1016/j.pneurobio.2020.101879 32615146

[B5] Bahri M. A. Plenevaux A. Aerts J. Bastin C. Becker G. Mercier J. (2017). Measuring brain synaptic vesicle protein 2A with positron emission tomography and [(18)F]UCB-H. *Alzheimers Dement.* 3 481–486. 10.1016/j.trci.2017.08.004 29124105 PMC5671624

[B6] Bajjalieh S. M. Frantz G. D. Weimann J. M. McConnell S. K. Scheller R. H. (1994). Differential expression of synaptic vesicle protein 2 (SV2) isoforms. *J. Neurosci.* 14 5223–5235. 10.1523/JNEUROSCI.14-09-05223.1994 8083732 PMC6577109

[B7] Bajjalieh S. M. Peterson K. Linial M. Scheller R. H. (1993). Brain contains two forms of synaptic vesicle protein 2. *Proc. Natl. Acad. Sci. U.S.A.* 90 2150–2154. 10.1073/pnas.90.6.2150 7681585 PMC46043

[B8] Bajjalieh S. M. Peterson K. Shinghal R. Scheller R. H. (1992). SV2, a brain synaptic vesicle protein homologous to bacterial transporters. *Science* 257 1271–1273. 10.1126/science.1519064 1519064

[B9] Bastin C. Bahri M. A. Meyer F. Manard M. Delhaye E. Plenevaux A. (2020). In vivo imaging of synaptic loss in Alzheimer’s disease with [18F]UCB-H positron emission tomography. *Eur. J. Nucl. Med. Mol. Imaging* 47 390–402. 10.1007/s00259-019-04461-x 31468182

[B10] Becker G. Warnier C. Serrano M. E. Bahri M. A. Mercier J. Lemaire C. (2017). Pharmacokinetic characterization of [(18)F]UCB-H PET radiopharmaceutical in the rat brain. *Mol. Pharm.* 14 2719–2725. 10.1021/acs.molpharmaceut.7b00235 28651055

[B11] Bertoglio D. Verhaeghe J. Miranda A. Kertesz I. Cybulska K. Korat Š (2020). Validation and noninvasive kinetic modeling of [(11)C]UCB-J PET imaging in mice. *J. Cereb. Blood Flow Metab.* 40 1351–1362. 10.1177/0271678X19864081 31307287 PMC7232782

[B12] Bertoglio D. Verhaeghe J. Wyffels L. Miranda A. Stroobants S. Mrzljak L. (2021). Synaptic vesicle glycoprotein 2A is affected in the CNS of Huntington’s Disease mice and post-mortem human HD brain. *J. Nucl. Med.* [Epub ahead of print]. 10.2967/jnumed.121.262709 34531262 PMC9157723

[B13] Binda K. H. Lillethorup T. P. Real C. C. Baerentzen S. L. Nielsen M. N. Orlowski D. (2021). Exercise protects synaptic density in a rat model of Parkinson’s disease. *Exp. Neurol.* 342:113741. 10.1016/j.expneurol.2021.113741 33965411

[B14] Bindra P. S. Knowles R. Buckley K. M. (1993). Conservation of the amino acid sequence of SV2, a transmembrane transporter in synaptic vesicles and endocrine cells. *Gene* 137 299–302. 10.1016/0378-1119(93)90024-w 8299963

[B15] Bini J. Holden D. Fontaine K. Mulnix T. Lu Y. Matuskey D. (2020). Human adult and adolescent biodistribution and dosimetry of the synaptic vesicle glycoprotein 2A radioligand (11)C-UCB-J. *EJNMMI Res.* 10:83. 10.1186/s13550-020-00670-w 32666239 PMC7359974

[B16] Blum F. C. Chen C. Kroken A. R. Barbieri J. T. (2012). Tetanus toxin and botulinum toxin a utilize unique mechanisms to enter neurons of the central nervous system. *Infect. Immun.* 80 1662–1669. 10.1128/IAI.00057-12 22392932 PMC3347426

[B17] Bradberry M. M. Chapman E. R. (2021). All-optical monitoring of excitation-secretion coupling demonstrates that SV2A functions downstream of evoked Ca(2+) entry. *J. Physiol.* 600 645–654. 10.1113/JP282601 34957569 PMC8810609

[B18] Bretin F. Bahri M. A. Bernard C. Warnock G. Aerts J. Mestdagh N. (2015). Biodistribution and radiation dosimetry for the novel SV2A radiotracer [(18)F]UCB-H: first-in-human study. *Mol. Imaging Biol.* 17 557–564. 10.1007/s11307-014-0820-6 25595813

[B19] Bretin F. Warnock G. Bahri M. A. Aerts J. Mestdagh N. Buchanan T. (2013). Preclinical radiation dosimetry for the novel SV2A radiotracer [18F]UCB-H. *EJNMMI Res.* 3:35. 10.1186/2191-219X-3-35 23647774 PMC3655042

[B20] Buckley K. Kelly R. B. (1985). Identification of a transmembrane glycoprotein specific for secretory vesicles of neural and endocrine cells. *J. Cell Biol.* 100 1284–1294. 10.1083/jcb.100.4.1284 2579958 PMC2113776

[B21] Budzinski K. L. Allen R. W. Fujimoto B. S. Kensel-Hammes P. Belnap D. M. Bajjalieh S. M. (2009). Large structural change in isolated synaptic vesicles upon loading with neurotransmitter. *Biophys. J.* 97 2577–2584. 10.1016/j.bpj.2009.08.032 19883601 PMC2770603

[B22] Cai H. Mangner T. J. Muzik O. Wang M. W. Chugani D. C. Chugani H. T. (2014). Radiosynthesis of (11)C-Levetiracetam: a potential marker for PET imaging of SV2A expression. *ACS Med. Chem. Lett.* 5 1152–1155. 10.1021/ml500285t 25313330 PMC4190623

[B23] Cai Z. Li S. Zhang W. Pracitto R. Wu X. Baum E. (2020). Synthesis and preclinical evaluation of an (18)F-Labeled synaptic vesicle glycoprotein 2A PET imaging probe: [(18)F]SynVesT-2. *ACS Chem. Neurosci.* 11 592–603. 10.1021/acschemneuro.9b00618 31961649

[B24] Chang W. P. Südhof T. C. (2009). SV2 renders primed synaptic vesicles competent for Ca2+ -induced exocytosis. *J. Neurosci.* 29 883–897. 10.1523/JNEUROSCI.4521-08.2009 19176798 PMC2693337

[B25] Chen M. K. Mecca A. P. Naganawa M. Finnema S. J. Toyonaga T. Lin S. (2018). Assessing synaptic density in Alzheimer Disease with synaptic vesicle glycoprotein 2A positron emission tomographic imaging. *JAMA Neurol.* 75 1215–1224. 10.1001/jamaneurol.2018.1836 30014145 PMC6233853

[B26] Chen M. K. Mecca A. P. Naganawa M. Gallezot J. D. Toyonaga T. Mondal J. (2021). Comparison of [(11)C]UCB-J and [(18)F]FDG PET in Alzheimer’s disease: a tracer kinetic modeling study. *J. Cereb. Blood Flow Metab.* 41 2395–2409. 10.1177/0271678X211004312 33757318 PMC8393289

[B27] Clare R. King V. G. Wirenfeldt M. Vinters H. V. (2010). Synapse loss in dementias. *J. Neurosci. Res.* 88 2083–2090. 10.1002/jnr.22392 20533377 PMC3068914

[B28] Colasante C. Rossetto O. Morbiato L. Pirazzini M. Molgó J. Montecucco C. (2013). Botulinum neurotoxin type A is internalized and translocated from small synaptic vesicles at the neuromuscular junction. *Mol. Neurobiol.* 48 120–127. 10.1007/s12035-013-8423-9 23471747

[B29] Constantinescu C. C. Tresse C. Zheng M. Gouasmat A. Carroll V. M. Mistico L. (2019). Development and *in Vivo* preclinical imaging of fluorine-18-Labeled synaptic vesicle protein 2A (SV2A) PET tracers. *Mol. Imaging Biol.* 21 509–518. 10.1007/s11307-018-1260-5 30084043

[B30] Contreras-García I. J. Gómez-Lira G. Phillips-Farfán B. V. Pichardo-Macías L. A. García-Cruz M. E. Chávez-Pacheco J. L. (2021). Synaptic vesicle protein 2A expression in glutamatergic terminals is associated with the response to levetiracetam treatment. *Brain Sci.* 11:531. 10.3390/brainsci11050531 33922424 PMC8145097

[B31] Contreras-García I. J. Pichardo-Macías L. A. Santana-Gómez C. E. Sánchez-Huerta K. Ramírez-Hernández R. Gómez-González B. (2018). Differential expression of synaptic vesicle protein 2A after status epilepticus and during epilepsy in a lithium-pilocarpine model. *Epilepsy Behav.* 88 283–294. 10.1016/j.yebeh.2018.08.023 30336420

[B32] Correa-Basurto J. Cuevas-Hernández R. I. Phillips-Farfán B. V. Martínez-Archundia M. Romo-Mancillas A. Ramírez-Salinas G. L. (2015). Identification of the antiepileptic racetam binding site in the synaptic vesicle protein 2A by molecular dynamics and docking simulations. *Front. Cell Neurosci.* 9:125. 10.3389/fncel.2015.00125 25914622 PMC4392693

[B33] Crèvecoeur J. Foerch P. Doupagne M. Thielen C. Vandenplas C. Moonen G. (2013). Expression of SV2 isoforms during rodent brain development. *BMC Neurosci.* 14:87. 10.1186/1471-2202-14-87 23937191 PMC3765414

[B34] Crowder K. M. Gunther J. M. Jones T. A. Hale B. D. Zhang H. Z. Peterson M. R. (1999). Abnormal neurotransmission in mice lacking synaptic vesicle protein 2A (SV2A). *Proc. Natl. Acad. Sci. U.S.A.* 96 15268–15273. 10.1073/pnas.96.26.15268 10611374 PMC24809

[B35] Custer K. L. Austin N. S. Sullivan J. M. Bajjalieh S. M. (2006). Synaptic vesicle protein 2 enhances release probability at quiescent synapses. *J. Neurosci.* 26 1303–1313. 10.1523/JNEUROSCI.2699-05.2006 16436618 PMC6674579

[B36] Dardou D. Dassesse D. Cuvelier L. Deprez T. De Ryck M. Schiffmann S. N. (2011). Distribution of SV2C mRNA and protein expression in the mouse brain with a particular emphasis on the basal ganglia system. *Brain Res.* 1367 130–145. 10.1016/j.brainres.2010.09.063 20869353

[B37] Delva A. Michiels L. Koole M. Van Laere K. Vandenberghe W. (2021). Synaptic damage and its clinical correlates in people with early Huntington disease: a PET study. *Neurology* 98 e83–e94. 10.1212/WNL.0000000000012969 34663644

[B38] Delva A. Van Weehaeghe D. Koole M. Van Laere K. Vandenberghe W. (2020). Loss of presynaptic terminal integrity in the substantia nigra in early Parkinson’s Disease. *Mov. Disord.* 35 1977–1986. 10.1002/mds.28216 32767618

[B39] Dong M. Liu H. Tepp W. H. Johnson E. A. Janz R. Chapman E. R. (2008). Glycosylated SV2A and SV2B mediate the entry of botulinum neurotoxin E into neurons. *Mol. Biol. Cell* 19 5226–5237. 10.1091/mbc.e08-07-0765 18815274 PMC2592654

[B40] Dong M. Yeh F. Tepp W. H. Dean C. Johnson E. A. Janz R. (2006). SV2 is the protein receptor for botulinum neurotoxin A. *Science* 312 592–596. 10.1126/science.1123654 16543415

[B41] Douaud M. Feve K. Pituello F. Gourichon D. Boitard S. Leguern E. (2011). Epilepsy caused by an abnormal alternative splicing with dosage effect of the SV2A gene in a chicken model. *PLoS One* 6:e26932. 10.1371/journal.pone.0026932 22046416 PMC3203167

[B42] D’Souza D. C. Radhakrishnan R. Naganawa M. Ganesh S. Nabulsi N. Najafzadeh S. (2021). Preliminary in vivo evidence of lower hippocampal synaptic density in cannabis use disorder. *Mol. Psychiatry* 26 3192–3200. 10.1038/s41380-020-00891-4 32973170

[B43] Estrada S. Lubberink M. Thibblin A. Sprycha M. Buchanan T. Mestdagh N. (2016). [(11)C]UCB-A, a novel PET tracer for synaptic vesicle protein 2A. *Nucl. Med. Biol.* 43 325–332. 10.1016/j.nucmedbio.2016.03.004 27260773

[B44] Feany M. B. Lee S. Edwards R. H. Buckley K. M. (1992). The synaptic vesicle protein SV2 is a novel type of transmembrane transporter. *Cell* 70 861–867. 10.1016/0092-8674(92)90319-8 1355409

[B45] Feng G. Xiao F. Lu Y. Huang Z. Yuan J. Xiao Z. (2009). Down-regulation synaptic vesicle protein 2A in the anterior temporal neocortex of patients with intractable epilepsy. *J. Mol. Neurosci.* 39 354–359. 10.1007/s12031-009-9288-2 19757204

[B46] Finnema S. J. Nabulsi N. B. Eid T. Detyniecki K. Lin S. F. Chen M. K. (2016). Imaging synaptic density in the living human brain. *Sci. Transl. Med.* 8:348ra96. 10.1126/scitranslmed.aaf6667 27440727

[B47] Finnema S. J. Nabulsi N. B. Mercier J. Lin S. F. Chen M. K. Matuskey D. (2018). Kinetic evaluation and test-retest reproducibility of [(11)C]UCB-J, a novel radioligand for positron emission tomography imaging of synaptic vesicle glycoprotein 2A in humans. *J. Cereb. Blood Flow Metab.* 38 2041–2052. 10.1177/0271678X17724947 28792356 PMC6259313

[B48] Finnema S. J. Rossano S. Naganawa M. Henry S. Gao H. Pracitto R. (2019). A single-center, open-label positron emission tomography study to evaluate brivaracetam and levetiracetam synaptic vesicle glycoprotein 2A binding in healthy volunteers. *Epilepsia* 60 958–967. 10.1111/epi.14701 30924924 PMC6532410

[B49] Finnema S. J. Toyonaga T. Detyniecki K. Chen M. K. Dias M. Wang Q. (2020). Reduced synaptic vesicle protein 2A binding in temporal lobe epilepsy: a [(11) C]UCB-J positron emission tomography study. *Epilepsia* 61 2183–2193. 10.1111/epi.16653 32944949

[B50] Fu Z. Chen C. Barbieri J. T. Kim J. J. Baldwin M. R. (2009). Glycosylated SV2 and gangliosides as dual receptors for botulinum neurotoxin serotype F. *Biochemistry* 48 5631–5641. 10.1021/bi9002138 19476346 PMC2709598

[B51] García-Pérez E. Mahfooz K. Covita J. Zandueta A. Wesseling J. F. (2015). Levetiracetam accelerates the onset of supply rate depression in synaptic vesicle trafficking. *Epilepsia* 56 535–545. 10.1111/epi.12930 25684406

[B52] Gillard M. Chatelain P. Fuks B. (2006). Binding characteristics of levetiracetam to synaptic vesicle protein 2A (SV2A) in human brain and in CHO cells expressing the human recombinant protein. *Eur. J. Pharmacol.* 536 102–108. 10.1016/j.ejphar.2006.02.022 16556440

[B53] Goutal S. Guillermier M. Becker G. Gaudin M. Bramoullé Y. Luxen A. (2021). The pharmacokinetics of [(18)F]UCB-H revisited in the healthy non-human primate brain. *EJNMMI Res.* 11: 36. 10.1186/s13550-021-00777-8 33826008 PMC8026785

[B54] Gower A. J. Noyer M. Verloes R. Gobert J. Wülfert E. (1992). ucb L059, a novel anti-convulsant drug: pharmacological profile in animals. *Eur. J. Pharmacol.* 222 193–203. 10.1016/0014-2999(92)90855-x 1451732

[B55] Groves P. M. Linder J. C. Young S. J. (1994). 5-hydroxydopamine-labeled dopaminergic axons: three-dimensional reconstructions of axons, synapses and postsynaptic targets in rat neostriatum. *Neuroscience* 58 593–604. 10.1016/0306-4522(94)90084-1 8170539

[B56] Gustafsson R. Zhang S. Masuyer G. Dong M. Stenmark P. (2018). Crystal structure of botulinum neurotoxin A2 in complex with the human protein receptor SV2C reveals plasticity in receptor binding. *Toxins* 10:153. 10.3390/toxins10040153 29649119 PMC5923319

[B57] Halff E. F. Cotel M. C. Natesan S. McQuade R. Ottley C. J. Srivastava D. P. (2021). Effects of chronic exposure to haloperidol, olanzapine or lithium on SV2A and NLGN synaptic puncta in the rat frontal cortex. *Behav. Brain Res.* 405:113203. 10.1016/j.bbr.2021.113203 33636238

[B58] Hanaya R. Hosoyama H. Sugata S. Tokudome M. Hirano H. Tokimura H. (2012). Low distribution of synaptic vesicle protein 2A and synaptotagimin-1 in the cerebral cortex and hippocampus of spontaneously epileptic rats exhibiting both tonic convulsion and absence seizure. *Neuroscience* 221 12–20. 10.1016/j.neuroscience.2012.06.058 22766234

[B59] Harada S. Tanaka S. Takahashi Y. Matsumura H. Shimamoto C. Nakano T. (2013). Inhibition of Ca(2+)-regulated exocytosis by levetiracetam, a ligand for SV2A, in antral mucous cells of guinea pigs. *Eur. J. Pharmacol.* 721 185–192. 10.1016/j.ejphar.2013.09.037 24076180

[B60] Harper C. B. Small C. Davenport E. C. Low D. W. Smillie K. J. Martínez-Mármol R. (2020). An Epilepsy-associated SV2A mutation disrupts synaptotagmin-1 expression and activity-dependent trafficking. *J. Neurosci.* 40 4586–4595. 10.1523/JNEUROSCI.0210-20.2020 32341095 PMC7275853

[B61] Heidegger T. Krakow K. Ziemann U. (2010). Effects of antiepileptic drugs on associative LTP-like plasticity in human motor cortex. *Eur. J. Neurosci.* 32 1215–1222. 10.1111/j.1460-9568.2010.07375.x 20726885

[B62] Holland N. Jones P. S. Savulich G. Wiggins J. K. Hong Y. T. Fryer T. D. (2020). Synaptic loss in primary tauopathies revealed by [(11) C]UCB-J positron emission tomography. *Mov. Disord.* 35 1834–1842. 10.1002/mds.28188 32652635 PMC7611123

[B63] Holmes S. E. Finnema S. J. Naganawa M. DellaGioia N. Holden D. Fowles K. (2022). Imaging the effect of ketamine on synaptic density (SV2A) in the living brain. *Mol. Psychiatry* [Epub ahead of print]. 10.1038/s41380-022-01465-2 35165397 PMC9133063

[B64] Holmes S. E. Scheinost D. Finnema S. J. Naganawa M. Davis M. T. DellaGioia N. (2019). Lower synaptic density is associated with depression severity and network alterations. *Nat. Commun.* 10:1529. 10.1038/s41467-019-09562-7 30948709 PMC6449365

[B65] Hung T. Y. Wu S. N. Huang C. W. (2021). The integrated effects of brivaracetam, a selective analog of levetiracetam, on ionic currents and neuronal excitability. *Biomedicines* 9:369. 10.3390/biomedicines9040369 33916190 PMC8067033

[B66] Iezzi M. Theander S. Janz R. Loze C. Wollheim C. B. (2005). SV2A and SV2C are not vesicular Ca2+ transporters but control glucose-evoked granule recruitment. *J. Cell Sci.* 118 5647–5660. 10.1242/jcs.02658 16306227

[B67] Jacobsson J. A. Haitina T. Lindblom J. Fredriksson R. (2007). Identification of six putative human transporters with structural similarity to the drug transporter SLC22 family. *Genomics* 90 595–609. 10.1016/j.ygeno.2007.03.017 17714910

[B68] Janz R. Goda Y. Geppert M. Missler M. Südhof T. C. (1999). SV2A and SV2B function as redundant Ca2+ regulators in neurotransmitter release. *Neuron* 24 1003–1016. 10.1016/s0896-6273(00)81046-6 10624962

[B69] Janz R. Südhof T. C. (1999). SV2C is a synaptic vesicle protein with an unusually restricted localization: anatomy of a synaptic vesicle protein family. *Neuroscience* 94 1279–1290. 10.1016/s0306-4522(99)00370-x 10625067

[B70] Ji-qun C. Ishihara K. Nagayama T. Serikawa T. Sasa M. (2005). Long-lasting antiepileptic effects of levetiracetam against epileptic seizures in the spontaneously epileptic rat (SER): differentiation of levetiracetam from conventional antiepileptic drugs. *Epilepsia* 46 1362–1370. 10.1111/j.1528-1167.2005.29604.x 16146430

[B71] Kaminski R. M. Gillard M. Leclercq K. Hanon E. Lorent G. Dassesse D. (2009). Proepileptic phenotype of SV2A-deficient mice is associated with reduced anticonvulsant efficacy of levetiracetam. *Epilepsia* 50 1729–1740. 10.1111/j.1528-1167.2009.02089.x 19486357

[B72] Klitgaard H. Matagne A. Gobert J. Wülfert E. (1998). Evidence for a unique profile of levetiracetam in rodent models of seizures and epilepsy. *Eur. J. Pharmacol.* 353 191–206. 10.1016/s0014-2999(98)00410-5 9726649

[B73] Koole M. van Aalst J. Devrome M. Mertens N. Serdons K. Lacroix B. (2019). Quantifying SV2A density and drug occupancy in the human brain using [(11)C]UCB-J PET imaging and subcortical white matter as reference tissue. *Eur. J. Nucl. Med. Mol. Imaging* 46 396–406. 10.1007/s00259-018-4119-8 30121895

[B74] Kroken A. R. Karalewitz A. P. Fu Z. Kim J. J. Barbieri J. T. (2011). Novel ganglioside-mediated entry of botulinum neurotoxin serotype D into neurons. *J. Biol. Chem.* 286 26828–26837. 10.1074/jbc.M111.254086 21632541 PMC3143643

[B75] Kwon S. E. Chapman E. R. (2012). Glycosylation is dispensable for sorting of synaptotagmin 1 but is critical for targeting of SV2 and synaptophysin to recycling synaptic vesicles. *J. Biol. Chem.* 287 35658–35668. 10.1074/jbc.M112.398883 22908222 PMC3471705

[B76] Lambeng N. Grossmann M. Chatelain P. Fuks B. (2006). Solubilization and immunopurification of rat brain synaptic vesicle protein 2A with maintained binding properties. *Neurosci. Lett.* 398 107–112. 10.1016/j.neulet.2005.12.059 16434140

[B77] Lazzell D. R. Belizaire R. Thakur P. Sherry D. M. Janz R. (2004). SV2B regulates synaptotagmin 1 by direct interaction. *J. Biol. Chem.* 279 52124–52131. 10.1074/jbc.M407502200 15466855

[B78] Lee J. Daniels V. Sands Z. A. Lebon F. Shi J. Biggin P. C. (2015). Exploring the interaction of SV2A with racetams using homology modelling, molecular dynamics and site-directed mutagenesis. *PLoS One* 10:e0116589. 10.1371/journal.pone.0116589 25692762 PMC4333566

[B79] Li M. Xue L. (2018). Levetiracetam inhibits endocytosis and augments short-term depression. *Pharmazie* 73 643–646. 10.1691/ph.2018.8634 30396382

[B80] Li S. Cai Z. Zhang W. Holden D. Lin S. F. Finnema S. J. (2019b). Synthesis and in vivo evaluation of [(18)F]UCB-J for PET imaging of synaptic vesicle glycoprotein 2A (SV2A). *Eur. J. Nucl. Med. Mol. Imaging* 46 1952–1965. 10.1007/s00259-019-04357-w 31175396 PMC6810698

[B81] Li S. Cai Z. Wu X. Holden D. Pracitto R. Kapinos M. (2019a). Synthesis and in Vivo evaluation of a novel PET radiotracer for imaging of synaptic vesicle glycoprotein 2A (SV2A) in Nonhuman Primates. *ACS Chem. Neurosci.* 10 1544–1554. 10.1021/acschemneuro.8b00526 30396272 PMC6810685

[B82] Li S. Naganawa M. Pracitto R. Najafzadeh S. Holden D. Henry S. (2021). Assessment of test-retest reproducibility of [(18)F]SynVesT-1, a novel radiotracer for PET imaging of synaptic vesicle glycoprotein 2A. *Eur. J. Nucl. Med. Mol. Imaging* 48 1327–1338. 10.1007/s00259-020-05149-3 33416954

[B83] Löscher W. Hönack D. (1993). Profile of ucb L059, a novel anticonvulsant drug, in models of partial and generalized epilepsy in mice and rats. *Eur. J. Pharmacol.* 232 147–158. 10.1016/0014-2999(93)90768-d 8467854

[B84] Lu Y. Toyonaga T. Naganawa M. Gallezot J. D. Chen M. K. Mecca A. P. (2021). Partial volume correction analysis for (11)C-UCB-J PET studies of Alzheimer’s disease. *Neuroimage* 238: 1182481. 10.1016/j.neuroimage.2021.118248 34119639 PMC8454285

[B85] Lynch B. A. Lambeng N. Nocka K. Kensel-Hammes P. Bajjalieh S. M. Matagne A. (2004). The synaptic vesicle protein SV2A is the binding site for the antiepileptic drug levetiracetam. *Proc. Natl. Acad. Sci. U.S.A.* 101 9861–9866. 10.1073/pnas.0308208101 15210974 PMC470764

[B86] Lynch B. A. Matagne A. Brännström A. von Euler A. Jansson M. Hauzenberger E. (2008). Visualization of SV2A conformations in situ by the use of Protein Tomography. *Biochem. Biophys. Res. Commun.* 375 491–495. 10.1016/j.bbrc.2008.07.145 18692481

[B87] Madeo M. Kovács A. D. Pearce D. A. (2014). The human synaptic vesicle protein, SV2A, functions as a galactose transporter in Saccharomyces cerevisiae. *J. Biol. Chem.* 289 33066–33071. 10.1074/jbc.C114.584516 25326386 PMC4246065

[B88] Mahrhold S. Strotmeier J. Garcia-Rodriguez C. Lou J. Marks J. D. Rummel A. (2013). Identification of the SV2 protein receptor-binding site of botulinum neurotoxin type E. *Biochem. J.* 453 37–47. 10.1042/BJ20130391 23621114

[B89] Mansur A. Rabiner E. A. Comley R. A. Lewis Y. Middleton L. T. Huiban M. (2020). Characterization of 3 PET tracers for quantification of mitochondrial and synaptic function in healthy human brain: (18)F-BCPP-EF, (11)C-SA-4503, and (11)C-UCB-J. *J. Nucl. Med.* 61 96–103. 10.2967/jnumed.119.228080 31324712

[B90] Margineanu D. G. Matagne A. Kaminski R. M. Klitgaard H. (2008). Effects of chronic treatment with levetiracetam on hippocampal field responses after pilocarpine-induced status epilepticus in rats. *Brain Res. Bull.* 77 282–285. 10.1016/j.brainresbull.2008.07.006 18722515

[B91] Matuskey D. Tinaz S. Wilcox K. C. Naganawa M. Toyonaga T. Dias M. (2020). Synaptic changes in parkinson disease assessed with in vivo imaging. *Ann. Neurol.* 87 329–338. 10.1002/ana.25682 31953875 PMC7065227

[B92] Mecca A. P. Chen M. K. O’Dell R. S. Naganawa M. Toyonaga T. Godek T. A. (2020). In vivo measurement of widespread synaptic loss in Alzheimer’s disease with SV2A PET. *Alzheimers Dement.* 16 974–982. 10.1002/alz.12097 32400950 PMC7383876

[B93] Mecca A. P. Chen M. K. O’Dell R. S. Naganawa M. Toyonaga T. Godek T. A. (2021). Association of entorhinal cortical tau deposition and hippocampal synaptic density in older individuals with normal cognition and early Alzheimer’s disease. *Neurobiol. Aging* 111 44–53. 10.1016/j.neurobiolaging.2021.11.004 34963063 PMC8761170

[B94] Mecca A. P. O’Dell R. S. Sharp E. S. Banks E. R. Bartlett H. H. Zhao W. (2022). Synaptic density and cognitive performance in Alzheimer’s disease: a PET imaging study with [(11) C]UCB-J. *Alzheimers Dement.* [Epub ahead of print]. 10.1002/alz.12582 35174954 PMC9381645

[B95] Meehan A. L. Yang X. McAdams B. D. Yuan L. Rothman S. M. (2011). A new mechanism for antiepileptic drug action: vesicular entry may mediate the effects of levetiracetam. *J. Neurophysiol.* 106 1227–1239. 10.1152/jn.00279.2011 21653714 PMC3174821

[B96] Meehan A. L. Yang X. Yuan L. L. Rothman S. M. (2012). Levetiracetam has an activity-dependent effect on inhibitory transmission. *Epilepsia* 53 469–476. 10.1111/j.1528-1167.2011.03392.x 22292611

[B97] Mendoza-Torreblanca J. G. García-Cruz M. E. Sánchez-Cruz I. Gomez-Gonzalez B. Juárez-Méndez S. Gómez-Lira G. (2019). Analysis of differential expression of synaptic vesicle protein 2A in the adult rat brain. *Neuroscience* 419 108–120. 10.1016/j.neuroscience.2019.09.004 31520710

[B98] Menten-Dedoyart C. Serrano M. Navacerrada E. Bartholome O. Sánchez Gil J. Neirinckx V. (2016). Development and validation of a new mouse model to investigate the role of SV2A in Epilepsy. *PLoS One* 11:e0166525. 10.1371/journal.pone.0166525 27861538 PMC5115750

[B99] Mercier J. Archen L. Bollu V. Carré S. Evrard Y. Jnoff E. (2014). Discovery of heterocyclic nonacetamide synaptic vesicle protein 2A (SV2A) ligands with single-digit nanomolar potency: opening avenues towards the first SV2A positron emission tomography (PET) ligands. *ChemMedChem* 9 693–698. 10.1002/cmdc.201300482 24446373

[B100] Mertens N. Maguire R. P. Serdons K. Lacroix B. Mercier J. Sciberras D. (2020). Validation of parametric methods for [(11)C]UCB-J PET imaging using subcortical white matter as reference tissue. *Mol. Imaging Biol.* 22 444–452. 10.1007/s11307-019-01387-6 31209780

[B101] Metaxas A. Thygesen C. Briting S. R. R. Landau A. M. Darvesh S. Finsen B. (2019). Increased inflammation and unchanged density of synaptic vesicle glycoprotein 2A (SV2A) in the postmortem frontal cortex of Alzheimer’s Disease Patients. *Front. Cell Neurosci.* 13:538. 10.3389/fncel.2019.00538 31866830 PMC6906198

[B102] Michiels L. Delva A. van Aalst J. Ceccarini J. Vandenberghe W. Vandenbulcke M. (2021a). Synaptic density in healthy human aging is not influenced by age or sex: a (11)C-UCB-J PET study. *Neuroimage* 232:117877. 10.1016/j.neuroimage.2021.117877 33639258

[B103] Michiels L. Mertens N. Thijs L. Radwan A. Sunaert S. Vandenbulcke M. (2021b). Changes in synaptic density in the subacute phase after ischemic stroke: A (11)C-UCB-J PET/MR study. *J. Cereb. Blood Flow Metab.* 42 303–314. 10.1177/0271678X211047759 34550834 PMC9122519

[B104] Milicevic Sephton S. Miklovicz T. Russell J. J. Doke A. Li L. Boros I. (2020). Automated radiosynthesis of [(11) C]UCB-J for imaging synaptic density by positron emission tomography. *J. Labelled Comp. Radiopharm.* 63 151–158. 10.1002/jlcr.3828 32027052 PMC7155065

[B105] Mutch S. A. Kensel-Hammes P. Gadd J. C. Fujimoto B. S. Allen R. W. Schiro P. G. (2011). Protein quantification at the single vesicle level reveals that a subset of synaptic vesicle proteins are trafficked with high precision. *J. Neurosci.* 31 1461–1470. 10.1523/JNEUROSCI.3805-10.2011 21273430 PMC3078718

[B106] Nabulsi N. B. Mercier J. Holden D. Carré S. Najafzadeh S. Vandergeten M. C. (2016). Synthesis and preclinical evaluation of 11C-UCB-J as a PET tracer for imaging the synaptic vesicle glycoprotein 2A in the brain. *J. Nucl. Med.* 57 777–784. 10.2967/jnumed.115.168179 26848175

[B107] Naganawa M. Gallezot J. D. Finnema S. J. Matuskey D. Mecca A. Nabulsi N. B. (2021a). Simplified quantification of (11)C-UCB-J PET evaluated in a large human cohort. *J. Nucl. Med.* 62 418–421. 10.2967/jnumed.120.243949 32646875 PMC8049341

[B108] Naganawa M. Li S. Nabulsi N. Henry S. Zheng M. Q. Pracitto R. (2021b). First-in-Human evaluation of (18)F-SynVesT-1, a radioligand for PET imaging of synaptic vesicle Glycoprotein 2A. *J. Nucl. Med.* 62 561–567. 10.2967/jnumed.120.249144 32859701 PMC8049363

[B109] Nicastro N. Holland N. Savulich G. Carter S. F. Mak E. Hong Y. T. (2020). (11)C-UCB-J synaptic PET and multimodal imaging in dementia with Lewy bodies. *Eur. J. Hybrid Imaging* 4:25. 10.1186/s41824-020-00093-9 33381679 PMC7752786

[B110] Nowack A. Malarkey E. B. Yao J. Bleckert A. Hill J. Bajjalieh S. M. (2011). Levetiracetam reverses synaptic deficits produced by overexpression of SV2A. *PLoS One* 6:e29560. 10.1371/journal.pone.0029560 22220214 PMC3248421

[B111] Nowack A. Yao J. Custer K. L. Bajjalieh S. M. (2010). SV2 regulates neurotransmitter release via multiple mechanisms. *Am. J. Physiol. Cell Physiol.* 299 C960–C967. 10.1152/ajpcell.00259.2010 20702688 PMC2980308

[B112] O’Dell R. S. Mecca A. P. Chen M. K. Naganawa M. Toyonaga T. Lu Y. (2021). Association of Aβ deposition and regional synaptic density in early Alzheimer’s disease: a PET imaging study with [(11)C]UCB-J. *Alzheimers Res. Ther.* 13:11. 10.1186/s13195-020-00742-y PMC7786921 33402201

[B113] Ohno Y. Ishihara S. Terada R. Kikuta M. Sofue N. Kawai Y. (2009). Preferential increase in the hippocampal synaptic vesicle protein 2A (SV2A) by pentylenetetrazole kindling. *Biochem. Biophys. Res. Commun.* 390 415–420. 10.1016/j.bbrc.2009.09.035 19751703

[B114] Ohno Y. Ishihara S. Terada R. Serikawa T. Sasa M. (2010). Antiepileptogenic and anticonvulsive actions of levetiracetam in a pentylenetetrazole kindling model. *Epilepsy Res.* 89 360–364. 10.1016/j.eplepsyres.2010.01.011 20138737

[B115] Onwordi E. C. Halff E. F. Whitehurst T. Mansur A. Cotel M. C. Wells L. (2020). Synaptic density marker SV2A is reduced in schizophrenia patients and unaffected by antipsychotics in rats. *Nat. Commun.* 11:246. 10.1038/s41467-019-14122-0 31937764 PMC6959348

[B116] Onwordi E. C. Whitehurst T. Mansur A. Statton B. Berry A. Quinlan M. (2021). The relationship between synaptic density marker SV2A, glutamate and N-acetyl aspartate levels in healthy volunteers and schizophrenia: a multimodal PET and magnetic resonance spectroscopy brain imaging study. *Transl. Psychiatry* 11:393. 10.1038/s41398-021-01515-3 34282130 PMC8290006

[B117] Patel S. Knight A. Krause S. Teceno T. Tresse C. Li S. (2020). Preclinical In Vitro and In Vivo characterization of synaptic vesicle 2A-Targeting compounds amenable to F-18 labeling as potential PET radioligands for imaging of synapse integrity. *Mol. Imaging Biol.* 22 832–841. 10.1007/s11307-019-01428-0 31728839

[B118] Peng L. Tepp W. H. Johnson E. A. Dong M. (2011). Botulinum neurotoxin D uses synaptic vesicle protein SV2 and gangliosides as receptors. *PLoS Pathog.* 7:e1002008. 10.1371/journal.ppat.1002008 21483489 PMC3068998

[B119] Portela-Gomes G. M. Lukinius A. Grimelius L. (2000). Synaptic vesicle protein 2, A new neuroendocrine cell marker. *Am. J. Pathol.* 157 1299–1309. 10.1016/S0002-9440(10)64645-7 11021834 PMC1850151

[B120] Pyle R. A. Schivell A. E. Hidaka H. Bajjalieh S. M. (2000). Phosphorylation of synaptic vesicle protein 2 modulates binding to synaptotagmin. *J. Biol. Chem.* 275 17195–17200. 10.1074/jbc.M000674200 10747945

[B121] Radhakrishnan R. Skosnik P. D. Ranganathan M. Naganawa M. Toyonaga T. Finnema S. (2021). In vivo evidence of lower synaptic vesicle density in schizophrenia. *Mol. Psychiatry* 26 7690–7698. 10.1038/s41380-021-01184-0 34135473

[B122] Rashed H. M. Shamma R. N. El-Sabagh H. A. (2018). Preparation of (99m)Tc-levetiracetam intranasal microemulsion as the first radiotracer for SPECT imaging of the Synaptic Vesicle Protein SV2A. *Eur. J. Pharm. Sci.* 121 29–33. 10.1016/j.ejps.2018.05.019 29787786

[B123] Raval N. R. Gudmundsen F. Juhl M. Andersen I. V. Speth N. Videbaek A. (2021a). Synaptic density and neuronal metabolic function measured by positron emission tomography in the unilateral 6-OHDA rat model of Parkinson’s Disease. *Front. Synaptic Neurosci.* 13:715811. 10.3389/fnsyn.2021.715811 34867258 PMC8636601

[B124] Raval N. R. Johansen A. Donovan L. L. Ros N. F. Ozenne B. Hansen H. D. (2021b). A single dose of psilocybin increases synaptic density and decreases 5-HT(2A) receptor density in the pig brain. *Int. J. Mol. Sci.* 22:835. 10.3390/ijms22020835 33467676 PMC7830000

[B125] Reigada D. Díez-Pérez I. Gorostiza P. Verdaguer A. Gómez de Aranda I. Pineda O. (2003). Control of neurotransmitter release by an internal gel matrix in synaptic vesicles. *Proc. Natl. Acad. Sci. U.S.A.* 100 3485–3490. 10.1073/pnas.0336914100 12629223 PMC152319

[B126] Rizzoli S. O. Betz W. J. (2005). Synaptic vesicle pools. *Nat. Rev. Neurosci.* 6 57–69. 15611727 10.1038/nrn1583

[B127] Rokka J. Schlein E. Eriksson J. (2019). Improved synthesis of SV2A targeting radiotracer [(11)C]UCB-J. *EJNMMI Radiopharm. Chem.* 4:30. 10.1186/s41181-019-0080-5 31784919 PMC6884603

[B128] Rossano S. Toyonaga T. Finnema S. J. Naganawa M. Lu Y. Nabulsi N. (2020). Assessment of a white matter reference region for (11)C-UCB-J PET quantification. *J. Cereb. Blood Flow Metab.* 40 1890–1901. 10.1177/0271678X19879230 31570041 PMC7446568

[B129] Rummel A. Häfner K. Mahrhold S. Darashchonak N. Holt M. Jahn R. (2009). Botulinum neurotoxins C, E and F bind gangliosides via a conserved binding site prior to stimulation-dependent uptake with botulinum neurotoxin F utilising the three isoforms of SV2 as second receptor. *J. Neurochem.* 110 1942–1954. 10.1111/j.1471-4159.2009.06298.x 19650874

[B130] Sadasivam P. Fang X. T. Toyonaga T. Lee S. Xu Y. Zheng M. Q. (2021). Quantification of SV2A binding in rodent brain using [(18)F]SynVesT-1 and PET Imaging. *Mol. Imaging Biol.* 23 372–381. 10.1007/s11307-020-01567-9 33258040 PMC8105262

[B131] Salmon E. Bahri M. A. Plenevaux A. Becker G. Seret A. Delhaye E. (2021). In vivo exploration of synaptic projections in frontotemporal dementia. *Sci. Rep.* 11:16092. 10.1038/s41598-021-95499-1 34373529 PMC8352914

[B132] Schivell A. E. Batchelor R. H. Bajjalieh S. M. (1996). Isoform-specific, calcium-regulated interaction of the synaptic vesicle proteins SV2 and synaptotagmin. *J. Biol. Chem.* 271 27770–27775. 10.1074/jbc.271.44.27770 8910372

[B133] Schivell A. E. Mochida S. Kensel-Hammes P. Custer K. L. Bajjalieh S. M. (2005). SV2A and SV2C contain a unique synaptotagmin-binding site. *Mol. Cell Neurosci.* 29 56–64. 10.1016/j.mcn.2004.12.011 15866046

[B134] Scranton T. W. Iwata M. Carlson S. S. (1993). The SV2 protein of synaptic vesicles is a keratan sulfate proteoglycan. *J. Neurochem.* 61 29–44. 10.1111/j.1471-4159.1993.tb03535.x 7685814

[B135] Serajee F. J. Huq A. M. (2015). Homozygous mutation in synaptic vesicle glycoprotein 2A gene results in intractable epilepsy, involuntary movements, microcephaly, and developmental and growth retardation. *Pediatr. Neurol.* 52 642–646.e1. 10.1016/j.pediatrneurol.2015.02.011 26002053

[B136] Serrano M. E. Bahri M. A. Becker G. Seret A. Germonpré C. Lemaire C. (2020). Exploring with [(18)F]UCB-H the in vivo variations in SV2A expression through the Kainic Acid Rat model of temporal lobe epilepsy. *Mol. Imaging Biol.* 22 1197–1207. 10.1007/s11307-020-01488-7 32206990 PMC7497718

[B137] Serrano M. E. Bahri M. A. Becker G. Seret A. Mievis F. Giacomelli F. (2019b). Quantification of [(18)F]UCB-H Binding in the Rat Brain: from kinetic modelling to standardised uptake value. *Mol. Imaging Biol.* 21 888–897. 10.1007/s11307-018-1301-0 30460626

[B138] Serrano M. E. Becker G. Bahri M. A. Seret A. Mestdagh N. Mercier J. (2019a). Evaluating the In Vivo specificity of [(18)F]UCB-H for the SV2A protein, compared with SV2B and SV2C in Rats Using microPET. *Molecules* 24:1705. 10.3390/molecules24091705 31052478 PMC6538996

[B139] Shi J. Anderson D. Lynch B. A. Castaigne J. G. Foerch P. Lebon F. (2011). Combining modelling and mutagenesis studies of synaptic vesicle protein 2A to identify a series of residues involved in racetam binding. *Biochem. Soc. Trans.* 39 1341–1347. 10.1042/BST0391341 21936812

[B140] Shi J. Zhou F. Wang L. K. Wu G. F. (2015). Synaptic vesicle protein2A decreases in amygdaloid-kindling pharmcoresistant epileptic rats. *J. Huazhong Univ. Sci. Technolog. Med. Sci.* 35 716–722. 10.1007/s11596-015-1496-0 26489628

[B141] Smart K. Liu H. Matuskey D. Chen M. K. Torres K. Nabulsi N. (2021). Binding of the synaptic vesicle radiotracer [(11)C]UCB-J is unchanged during functional brain activation using a visual stimulation task. *J. Cereb. Blood Flow Metab.* 41 1067–1079. 10.1177/0271678X20946198 32757741 PMC8054713

[B142] Son Y. J. Scranton T. W. Sunderland W. J. Baek S. J. Miner J. H. Sanes J. R. (2000). The synaptic vesicle protein SV2 is complexed with an alpha5-containing laminin on the nerve terminal surface. *J. Biol. Chem.* 275 451–460. 10.1074/jbc.275.1.451 10617638

[B143] Stockburger C. Miano D. Baeumlisberger M. Pallas T. Arrey T. N. Karas M. (2016). A Mitochondrial Role of SV2a protein in aging and Alzheimer’s Disease: studies with levetiracetam. *J. Alzheimers Dis.* 50 201–215. 10.3233/JAD-150687 26639968

[B144] Stokholm K. Thomsen M. B. Phan J. A. Møller L. K. Bay-Richter C. Christiansen S. H. (2021). α-Synuclein overexpression increases dopamine D2/3 receptor binding and immune activation in a model of early Parkinson’s Disease. *Biomedicines* 9:1876. 10.3390/biomedicines9121876 34944691 PMC8698691

[B145] Takamori S. Holt M. Stenius K. Lemke E. A. Grønborg M. Riedel D. (2006). Molecular anatomy of a trafficking organelle. *Cell* 127 831–846. 10.1016/j.cell.2006.10.030 17110340

[B146] Talos D. M. Chang M. Kosaras B. Fitzgerald E. Murphy A. Folkerth R. D. (2013). Antiepileptic effects of levetiracetam in a rodent neonatal seizure model. *Pediatr. Res.* 73 24–30. 10.1038/pr.2012.151 23138400 PMC6745697

[B147] Thomsen M. B. Jacobsen J. Lillethorup T. P. Schacht A. C. Simonsen M. Romero-Ramos M. (2021b). In vivo imaging of synaptic SV2A protein density in healthy and striatal-lesioned rats with [11C]UCB-J PET. *J. Cereb. Blood Flow Metab.* 41 819–830. 10.1177/0271678X20931140 32538280 PMC7983510

[B148] Thomsen M. B. Ferreira S. A. Schacht A. C. Jacobsen J. Simonsen M. Betzer C. (2021a). PET imaging reveals early and progressive dopaminergic deficits after intra-striatal injection of preformed alpha-synuclein fibrils in rats. *Neurobiol. Dis.* 149:105229. 10.1016/j.nbd.2020.105229 33352233

[B149] Thomsen M. B. Schacht A. C. Alstrup A. K. O. Jacobsen J. Lillethorup T. P. Baerentzen S. L. (2020). Preclinical PET Studies of [(11)C]UCB-J Binding in Minipig Brain. *Mol. Imaging Biol.* 22 1290–1300. 10.1007/s11307-020-01506-8 32514885

[B150] Tokudome K. Okumura T. Shimizu S. Mashimo T. Takizawa A. Serikawa T. (2016a). Synaptic vesicle glycoprotein 2A (SV2A) regulates kindling epileptogenesis via GABAergic neurotransmission. *Sci. Rep.* 6:27420. 10.1038/srep27420 27265781 PMC4893657

[B151] Tokudome K. Okumura T. Terada R. Shimizu S. Kunisawa N. Mashimo T. (2016b). A missense mutation of the gene encoding synaptic vesicle glycoprotein 2A (SV2A) confers seizure susceptibility by disrupting amygdalar synaptic GABA release. *Front. Pharmacol.* 7:210. 10.3389/fphar.2016.0021 PMC4943941 27471467

[B152] Toyonaga T. Smith L. M. Finnema S. J. Gallezot J. D. Naganawa M. Bini J. (2019). In Vivo synaptic density imaging with (11)C-UCB-J detects treatment effects of saracatinib in a mouse model of Alzheimer Disease. *J. Nucl. Med.* 60 1780–1786. 10.2967/jnumed.118.223867 31101744 PMC6894376

[B153] Uchigashima M. Ohtsuka T. Kobayashi K. Watanabe M. (2016). Dopamine synapse is a neuroligin-2-mediated contact between dopaminergic presynaptic and GABAergic postsynaptic structures. *Proc. Natl. Acad. Sci. U.S.A.* 113 4206–4211. 10.1073/pnas.1514074113 27035941 PMC4839454

[B154] van Vliet E. A. Aronica E. Redeker S. Boer K. Gorter J. A. (2009). Decreased expression of synaptic vesicle protein 2A, the binding site for levetiracetam, during epileptogenesis and chronic epilepsy. *Epilepsia* 50 422–433. 10.1111/j.1528-1167.2008.01727.x 18717715

[B155] Vanoye-Carlo A. Gómez-Lira G. (2019). Differential expression of SV2A in hippocampal glutamatergic and GABAergic terminals during postnatal development. *Brain Res.* 1715 73–83. 10.1016/j.brainres.2019.03.021 30905653

[B156] Varnäs K. Stepanov V. Halldin C. (2020). Autoradiographic mapping of synaptic vesicle glycoprotein 2A in non-human primate and human brain. *Synapse* 74:e22157. 10.1002/syn.22157 32259300

[B157] Venkatesan K. Alix P. Marquet A. Doupagne M. Niespodziany I. Rogister B. (2012). Altered balance between excitatory and inhibitory inputs onto CA1 pyramidal neurons from SV2A-deficient but not SV2B-deficient mice. *J. Neurosci. Res.* 90 2317–2327. 10.1002/jnr.23111 22847229

[B158] Vogl C. Mochida S. Wolff C. Whalley B. J. Stephens G. J. (2012). The synaptic vesicle glycoprotein 2A ligand levetiracetam inhibits presynaptic Ca2+ channels through an intracellular pathway. *Mol. Pharmacol.* 82 199–208. 10.1124/mol.111.076687 22554805

[B159] Vogl C. Tanifuji S. Danis B. Daniels V. Foerch P. Wolff C. (2015). Synaptic vesicle glycoprotein 2A modulates vesicular release and calcium channel function at peripheral sympathetic synapses. *Eur. J. Neurosci.* 41 398–409. 10.1111/ejn.12799 25484265

[B160] Wang L. Shi J. Wu G. Zhou F. Hong Z. (2014). Hippocampal low-frequency stimulation increased SV2A expression and inhibited the seizure degree in pharmacoresistant amygdala-kindling epileptic rats. *Epilepsy Res.* 108 1483–1491. 10.1016/j.eplepsyres.2014.07.005 25205164

[B161] Wang M. M. Janz R. Belizaire R. Frishman L. J. Sherry D. M. (2003). Differential distribution and developmental expression of synaptic vesicle protein 2 isoforms in the mouse retina. *J. Comp. Neurol.* 460 106–122. 10.1002/cne.10636 12687700

[B162] Warnier C. Lemaire C. Becker G. Zaragoza G. Giacomelli F. Aerts J. (2016). Enabling efficient Positron Emission Tomography (PET) imaging of synaptic vesicle Glycoprotein 2A (SV2A) with a robust and one-step radiosynthesis of a highly potent (18)F-Labeled ligand ([(18)F]UCB-H). *J. Med. Chem.* 59 8955–8966. 10.1021/acs.jmedchem.6b00905 27598384

[B163] Warnock G. I. Aerts J. Bahri M. A. Bretin F. Lemaire C. Giacomelli F. (2014). Evaluation of 18F-UCB-H as a novel PET tracer for synaptic vesicle protein 2A in the brain. *J. Nucl. Med.* 55 1336–1341. 10.2967/jnumed.113.136143 24935992

[B164] Weisemann J. Stern D. Mahrhold S. Dorner B. G. Rummel A. (2016). Botulinum neurotoxin serotype a recognizes its protein receptor SV2 by a different mechanism than botulinum neurotoxin B Synaptotagmin. *Toxins* 8:154. 10.3390/toxins8050154 27196927 PMC4885069

[B165] Weiss J. J. Calvi R. Naganawa M. Toyonaga T. Farhadian S. F. Chintanaphol M. (2021). Preliminary In Vivo evidence of reduced synaptic density in Human Immunodeficiency Virus (HIV) despite antiretroviral therapy. *Clin. Infect. Dis.* 73 1404–1411. 10.1093/cid/ciab484 34050746 PMC8528400

[B166] Wilson H. Pagano G. de Natale E. R. Mansur A. Caminiti S. P. Polychronis S. (2020). Mitochondrial Complex 1, Sigma 1, and synaptic vesicle 2A in early drug-naive Parkinson’s Disease. *Mov. Disord.* 35 1416–1427. 10.1002/mds.28064 32347983

[B167] Wittig S. Ganzella M. Barth M. Kostmann S. Riedel D. Pérez-Lara Á (2021). Cross-linking mass spectrometry uncovers protein interactions and functional assemblies in synaptic vesicle membranes. *Nat. Commun.* 12:858. 10.1038/s41467-021-21102-w 33558502 PMC7870876

[B168] Xiong M. Roshanbin S. Rokka J. Schlein E. Ingelsson M. Sehlin D. (2021). In vivo imaging of synaptic density with [(11)C]UCB-J PET in two mouse models of neurodegenerative disease. *Neuroimage* 239:118302. 10.1016/j.neuroimage.2021.118302 34174391

[B169] Xu T. Bajjalieh S. M. (2001). SV2 modulates the size of the readily releasable pool of secretory vesicles. *Nat. Cell Biol.* 3 691–698. 10.1038/35087000 11483953

[B170] Yang X. F. Weisenfeld A. Rothman S. M. (2007). Prolonged exposure to levetiracetam reveals a presynaptic effect on neurotransmission. *Epilepsia* 48 1861–1869. 10.1111/j.1528-1167.2006.01132.x 17521346

[B171] Yao G. Zhang S. Mahrhold S. Lam K. H. Stern D. Bagramyan K. (2016). N-linked glycosylation of SV2 is required for binding and uptake of botulinum neurotoxin A. *Nat. Struct. Mol. Biol.* 23 656–662. 10.1038/nsmb.3245 27294781 PMC5033645

[B172] Yao J. Bajjalieh S. M. (2008). Synaptic vesicle protein 2 binds adenine nucleotides. *J. Biol. Chem.* 283 20628–20634. 10.1074/jbc.M800738200 18524768 PMC2475693

[B173] Yao J. Nowack A. Kensel-Hammes P. Gardner R. G. Bajjalieh S. M. (2010). Cotrafficking of SV2 and synaptotagmin at the synapse. *J. Neurosci.* 30 5569–5578. 10.1523/JNEUROSCI.4781-09.2010 20410110 PMC2866018

[B174] Yeh F. L. Dong M. Yao J. Tepp W. H. Lin G. Johnson E. A. (2010). SV2 mediates entry of tetanus neurotoxin into central neurons. *PLoS Pathog.* 6:e1001207. 10.1371/journal.ppat.1001207 21124874 PMC2991259

[B175] Zhang N. Gordon S. L. Fritsch M. J. Esoof N. Campbell D. G. Gourlay R. (2015). Phosphorylation of synaptic vesicle protein 2A at Thr84 by casein kinase 1 family kinases controls the specific retrieval of synaptotagmin-1. *J. Neurosci.* 35 2492–2507. 10.1523/JNEUROSCI.4248-14.2015 25673844 PMC4323530

[B176] Zheng C. Holden D. Zheng M. Q. Pracitto R. Wilcox K. C. Lindemann M. (2021). A metabolically stable PET tracer for imaging synaptic vesicle protein 2A: synthesis and preclinical characterization of [(18)F]SDM-16. *Eur. J. Nucl. Med. Mol. Imaging* 49 1482–1496. 10.1007/s00259-021-05597-5 34761284 PMC8940841

